# Plasma DNMT1 Activity for Assessing Tumor Burden and Predicting Neoadjuvant Therapy Response in Breast Cancer

**DOI:** 10.1002/advs.202501064

**Published:** 2025-05-02

**Authors:** Yingran Wang, Guozhi Zhang, Zhizhao Zhang, Mengsi Zhang, Jiao Chen, Ke Wang, Lu Liu, Jing Bao, Ming Chen, Xiaowei Qi, Mingxuan Gao

**Affiliations:** ^1^ Department of Clinical Laboratory Medicine Southwest Hospital Third Military Medical University (Army Medical University) Chongqing 400038 P. R. China; ^2^ Department of Breast and Thyroid Surgery Southwest Hospital Third Military Medical University (Army Medical University) Chongqing 400038 P. R. China; ^3^ Institute of Pathology and Southwest Cancer Center Southwest Hospital Third Military Medical University (Army Medical University) Chongqing 400038 P. R. China

**Keywords:** breast cancer, modularized tandem reaction, neoadjuvant therapy response prediction, plasma DNMT1, tumor burden assessment

## Abstract

DNA methylation is mediated by DNA methyltransferases (DNMTs), and the stability of their activity is essential for cellular fate. DNMT1 is considered one of the most promising targets for research. However, current detection techniques are limited in accurately quantifying its activity in peripheral blood. Here, a reaction system is developed known as DNMT1 Identification by Variable Activity (DIVA) for the highly sensitive detection of DNMT1 activity in the peripheral blood of breast cancer patients. DIVA can detect DNMT1 at levels as low as 10^−7^ U mL^−1^, with minimal time and cost. This method is applied to analyze 271 clinical samples, successfully evaluating tumor burden in patients staged I‐IV. Finally, this method is utilized to assess the prognosis of 22 patients undergoing neoadjuvant therapy, demonstrating good consistency with ultrasound imaging results. It is believed that DIVA could serve as an effective auxiliary technique for both the early detection of breast cancer and evaluation of neoadjuvant therapy.

## Graphical abstract

In this work, a detection method is presented for the activity of plasma DNA methyltransferase 1 (DNMT1), namely DNMT1 Identification by Variable Activity (DIVA). DIVA can detect plasma DNMT1 at levels as low as 10^−7^ U mL^−1^, also it successfully evaluated tumor burden and predicted the neoadjuvant therapy responses of breast cancer patients.

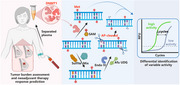

## 1 Introduction

Breast cancer, which has the highest incidence rate of malignancies among women,^[^ ^1^, ^2^ ^]^ has become a major threat to female and public health system. After decades of exploration into the diagnostic and treatment of breast cancer, advanced technologies in neoadjuvant therapy, surgery, adjuvant therapy, and radiotherapy have been developed.^[^ ^3^, ^4^, ^5^, ^6^, ^7^ ^]^ Especially neoadjuvant therapy has become a standard‐of‐care for patients with locally advanced breast cancer.^[^ ^8^, ^9^ ^]^ However, the use of liquid biopsy markers for breast cancer treatment response prediction and tumor burden assessment remains to be explored.^[^ ^10^, ^11^ ^]^ Epigenetic inheritance has a significant influence on the development of tumorigenesis, and DNA methyltransferases (DNMTs), which regulate epigenetics through DNA hypermethylation, are widely considered one of the important causes of breast cancer.^[^ ^12^, ^13^ ^]^ Substantial evidence shows that epigenetic modifications, including hypomethylation or hypermethylation of DNA, are involved in the occurrence and development of breast cancer^[^ ^14^, ^15^ ^]^ and the stability of DNMTs’ activity plays a vital physiological role in growth, development, genome stability, and cell fate.^[^ ^16^, ^17^ ^]^

The human DNMTs family primarily includes DNMT1, DNMT3A, and DNMT3B, which collectively perform the entire process of genome methylation.^[^ ^18^ ^]^ Among them, DNMT1 restores specific methylation patterns to the hemimethylated strand during replication based on the methylation patterns of the parent DNA, and its overexpression in all subtypes of breast cancer can lead to hypermethylation and carcinogenic activation.^[^ ^19^, ^20^ ^]^ Meanwhile, DNMT1 presented other specific oncogenic roles, such as repression of estrogen receptor,^[^ ^21^ ^]^ promotion of epithelial‐mesenchymal transition required for metastasis,^[^ ^22^ ^]^ inducing cellular autophagy,^[^ ^23^ ^]^ and promoting the growth of cancer stem cells.^[^ ^24^ ^]^ Compared to the changes in tumor phenotype (growth, migration, invasion, etc.), gene epigenetic changes that are mediated by DNMTs occur upstream in the timeline and may provide better early‐warning capacity. Therefore, we hypothesize that detecting DNMT1 could enable early warning and prognosis prediction for breast cancer.

However, current clinical detection methods for DNMT1 primarily rely on enzyme‐linked immunosorbent assay (ELISA) or immunohistochemistry, which may not be suitable for detecting DNMT1 in plasma at specific time points. ELISA has relatively limited sensitivity and is unable to quantify DNMT1 enzymatic activity in plasma. Pathological immunohistochemistry (IHC) requires tissue puncture, which poses a significant burden on patients and cannot effectively account for tissue morphology variations or possible heterogeneous effects caused by differences in phenotype and biological behavior. Researchers have attempted to develop more sensitive methods for the DNMT1 detection.^[^ ^25^, ^26^ ^]^ Xiao et al. developed a high‐throughput‐methyl‐reading assay detecting 20 nm DNMT1 by fluorescence polarization.^[^ ^27^ ^]^ Guo and co‐workers used an exponential amplification strategy to achieve DNMT1 detection as low as 0.009 U mL^−1^.^[^ ^28^ ^]^ However, these reported methods remain incapable of distinguishing different DNMT1 levels in human plasma. Therefore, establishing an effective detection method for plasma DNMT1, which can convert single baseline static prediction into an early dynamic assessment during diagnosis and treatment, is of great importance for the prognosis and survival of breast cancer patients.

**Scheme 1:**
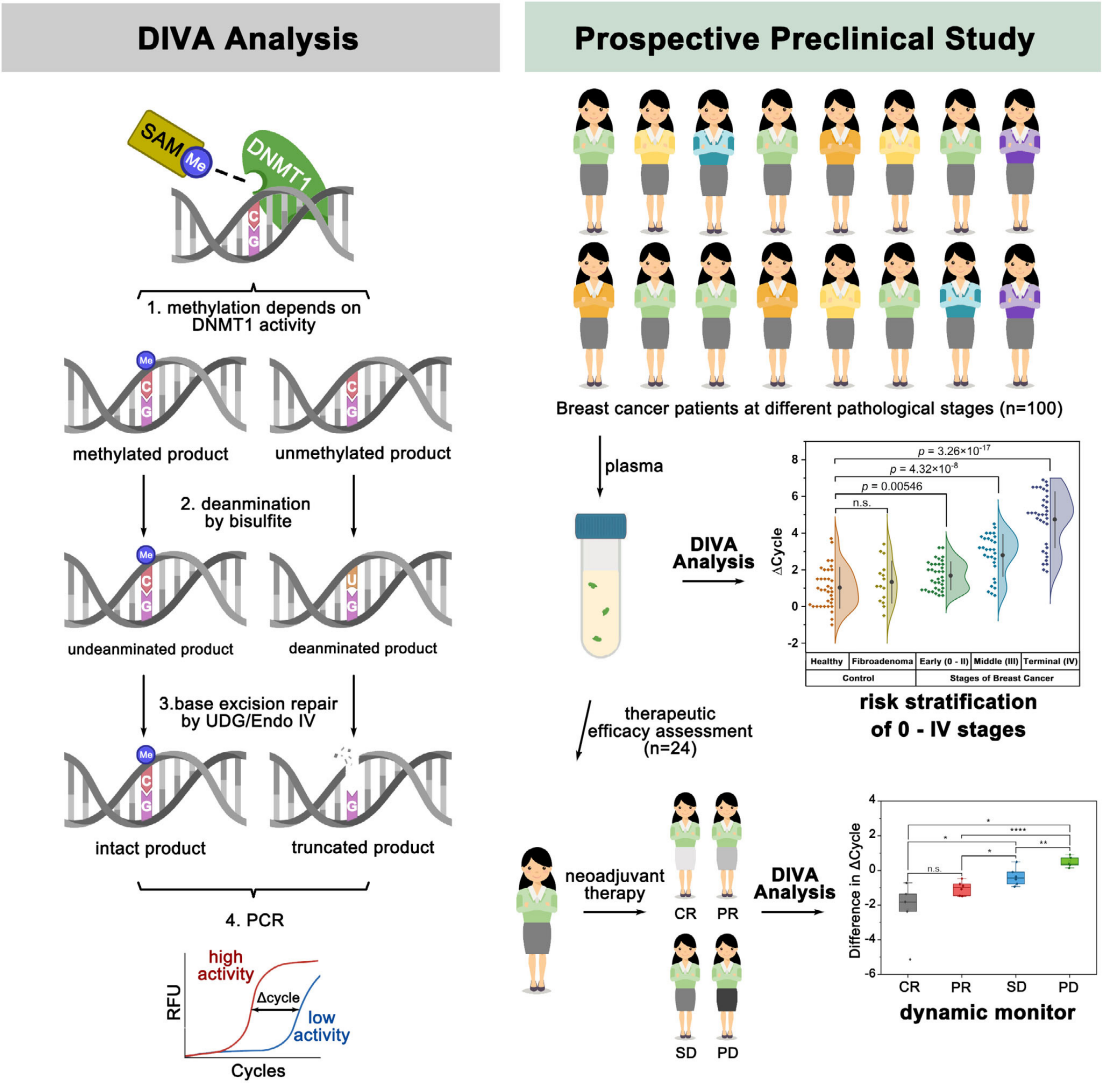
Illustration of the Differential Identification of Variable Activity (DIVA) system of DNMT1 detection for breast cancer patients.

In this work, we developed a modularized tandem reaction system called DIVA (DNMT1 Identification by Variable Activity) for the sensitive detection of DNMT1 in human plasma (Scheme 1). DIVA comprises three inter‐linked but independent modules: DNA methylation, cytosine deamination, and truncation. In this system, we designed a double‐stranded DNA (dsDNA) sequence with two staggered hemimethylated CpG positions in the middle of substrates for the DNMT1 reaction, while all remaining cytosines were fully methylated. In the first module, the DNMT1 distinguishes the hemimethylated CpG and methylates the cytosine; however, partial substrates remain unmethylated due to the DNMT1 activity. The product then undergoes deamination by bisulfite, where each unmethylated cytosine is transferred to uracil while leaving methylated cytosine intact;^[^ ^29^ ^]^ subsequently, the uracil is truncated in the third module by the *Afu* Uracil‐DNA Glycosylase (*Afu* UDG) and endonuclease IV (endo IV).^[^ ^30^, ^31^, ^32^ ^]^ The differential activity of DNMT1 is successfully translated into variations in dsDNA concentration, enabling efficient and rapid discrimination through PCR for accurate identification. DIVA can detect DNMT1 as low as 10^−7^ U mL^−1^. This sensitivity is sufficient for direct detection in plasma samples from breast cancer patients, an achievement not previously reported. The detection duration of DIVA is ≈6 h (from sample acquisition to obtaining results) and imposes less burden on patients, representing a significant improvement compared to IHC analysis (usually about 6 days).^[^ ^33^ ^]^ Through collaboration with our hospital's department of breast and thyroid surgery, we conducted a prospective clinical study using DIVA, successfully assessing tumor burden in breast cancer patients from stage I to IV. Further investigation revealed potential secretory sources of DNMT1 through immunohistochemical analysis. Additionally, DIVA effectively monitored the efficacy of neoadjuvant therapy, showing results consistent with ultrasound findings. This DIVA method opens a new perspective for using plasma DNMT1 as a monitoring indicator in the clinical tumor burden assessment and neoadjuvant therapy response prediction of breast cancer.

## 2 Results

### 2.1 Characterization and Examination of the DIVA System for DNMT1 Detection

**Figure 1:**
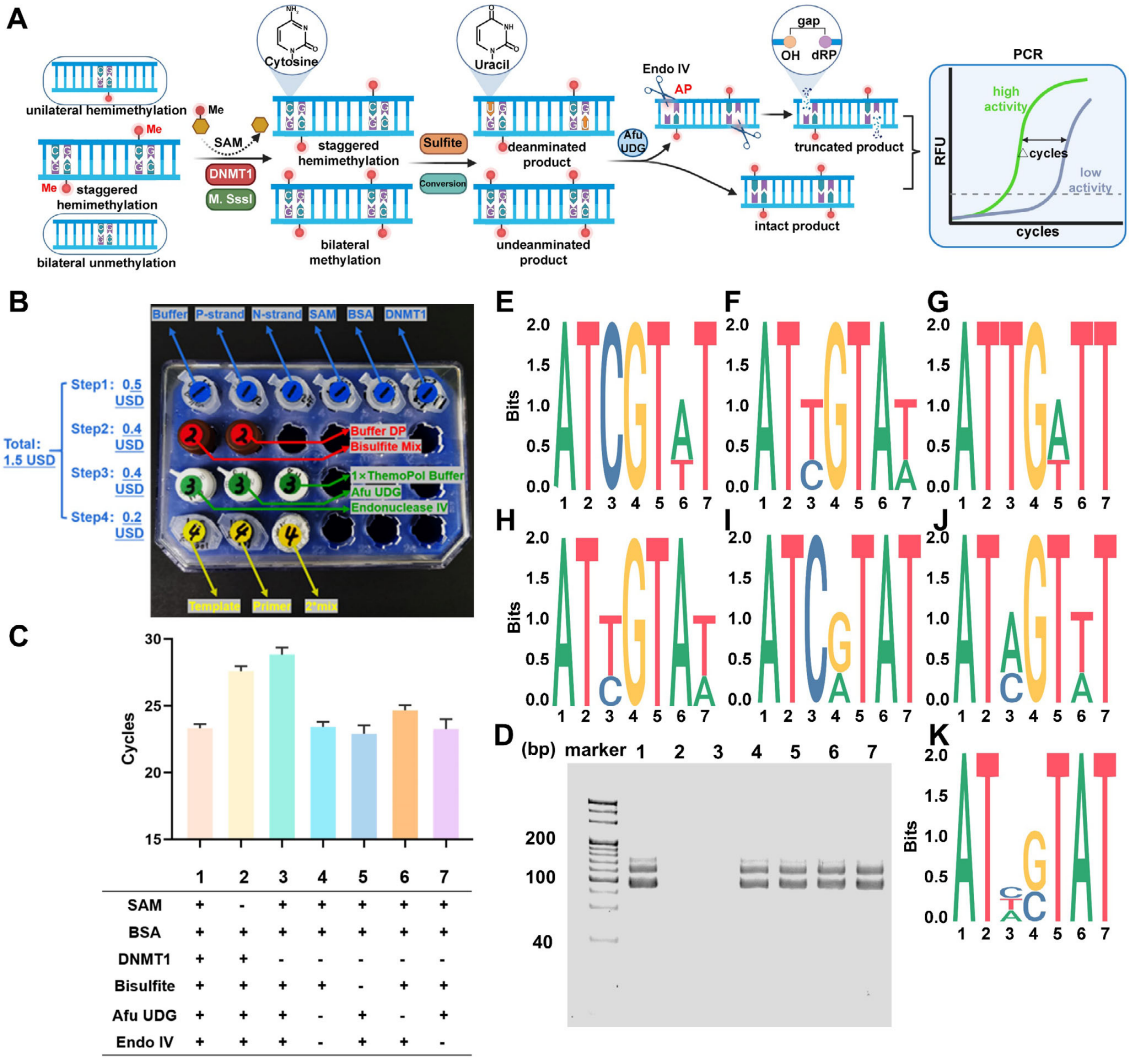
Characterization of the DIVA system for DNMT1 detection. A) Flowchart for the design of unilateral hemimethylated, staggered hemimethylated and bilateral unmethylated sequences for the detection of DNMT1 and M.SssI. B) Price chart for completing one experiment per sample. C) The column diagram of qPCR results for the different combination of substrates. *C*_DNA substrate_ = 250 fm, *C*_primer_ = 10 µm, *C*_SAM_ = 1.6 mm, *C*_DNMT1_ = 2×10^−3^ U mL^−1^, *C*_afu UDG_ = 10 U mL^−1^, *C*_endo IV_ = 50 U mL^−1^, data presented: Mean ± SD. D) Denatured SDS‐PAGE photographs of 15 cycles of qPCR products. E–K) TA clone sequencing results identified the cytosine to thymine changes for the different combination of substrates corresponding to Figure 1C.

Since DNMT1 methylates cytosine residues in hemimethylated CpG positions of dsDNA,^[^ ^34^, ^35^ ^]^ we designed the substrate sequence of DIVA to ensure two key features: 1) only the target site contains CpG for subsequent reactions, and 2) the forward and reverse strands of the substrates can hybridize into a perfectly matched duplex without forming dimers or other secondary structures. We included methylated cytosine only in the forward strand, using it to separate the adenine – thymine fragments. Guanine separated the thymine – adenine fragments in the reverse strand. The CpGs for DNMT1 detection were embedded in the middle of both strands, with methylated CpGs located at the corresponding positions on the other strand, forming a staggered hemimethylated structure. We also designed similar sequences with unilateral hemimethylation and bilateral unmethylation for the detection of hemimethylated enzymes and *de novo* methylated enzymes (**Figure** **1**A). To optimize amplification in the PCR reaction, we first examined the length of the substrates. The amplification and melting temperature curves with different concentrations of substrates (Figure S1A,B, Supporting Information) or primers (Figure S1C,D, Supporting Information) indicated specific amplification of substrate with 87nt and primer with 21nt. Denature polyacrylamide gel electrophoresis (PAGE, Figure S1E, Supporting Information) further confirmed that no nonspecific amplification or primer dimerization occurred. Although longer substrates and primers could promote the specificity during the PCR, our results showed that the as‐designed sequences could perform the reaction with high performance‐to‐price ratio, at less than 1.5 USD per sample (Figure 1B; see Table S1, Supporting Information for details).

The feasibility of this method was examined by both PCR and PAGE. With all reagents present, the designed CpG position was partially methylated based on DNMT1 activity. The methylated cytosine was not converted into uridine by the bisulfite, and thus could not be cleaved by *afu* UDG and endo IV in a base excision repair (BER) manner. Consequently, fewer PCR cycles (23.33 ± 0.29) were needed to amplify the uncleaved substrates with higher DNMT1 activity (2×10^−3^ U mL^−1^, Column 1, Figure 1C). In contrast, when DNMT1 was absent, the unmethylated cytosine remained and was converted into uridine and cleaved in the subsequent BER process, requiring more cycles (29.01 ± 0.47) PCR amplification (Column 3, Figure 1C). As S‐adenosylmethionine (SAM) supplies the methyl group, its absence also resulted in unmethylated cytosine and more cycles (27.58 ± 0.38, Column 2, Figure 1C). The lack of bisulfite, *afu* UDG, or endo IV hindered the successful cleavage of unmethylated cytosine even without DNMT1, resulting in fewer cycles (Column 4 – 7, Figure 1C).

These results were confirmed by the denatured PAGE. After 15 PCR cycles, the electrophoresis indicated the same results as the cycle numbers diagram. Lanes 2 and 3 showed no significant amplified bands due to the absence of DNMT1 or SAM. In other lanes (lanes 4 – 7), the unmethylated cytosine remained intact and amplified bands were detected. (Figure 1D). We used TA clone sequencing to directly identify the cytosine to thymine changes in our DIVA system and to verify the successful conversion of each sample (Figure S2, Supporting Information). With 2 × 10^−3^ U mL^−1^ DNMT1, the cytosine was fully methylated in the substrate and could not be converted into thymine (Figure 1E). However, in the absence of SAM or DNMT1, no methylation occurred and cytosine was eventually converted into thymine (Figure 1F,G). The deamination by bisulfite was confirmed to be efficient for the cytosine to uracil conversion (Figure 1H). When both methylation and deamination were absent, uracil formation could not occur and cytosine remained (Figure 1I). However, once uracil was generated, the *afu* UDG or Endo IV affected the sequencing readout of the original cytosine (Figure 1J,K). These results confirmed the necessity of each step in DIVA and demonstrated the mechanism for conversion and truncation.

### 2.2 Optimization of the Experimental Conditions of DIVA

**Figure 2:**
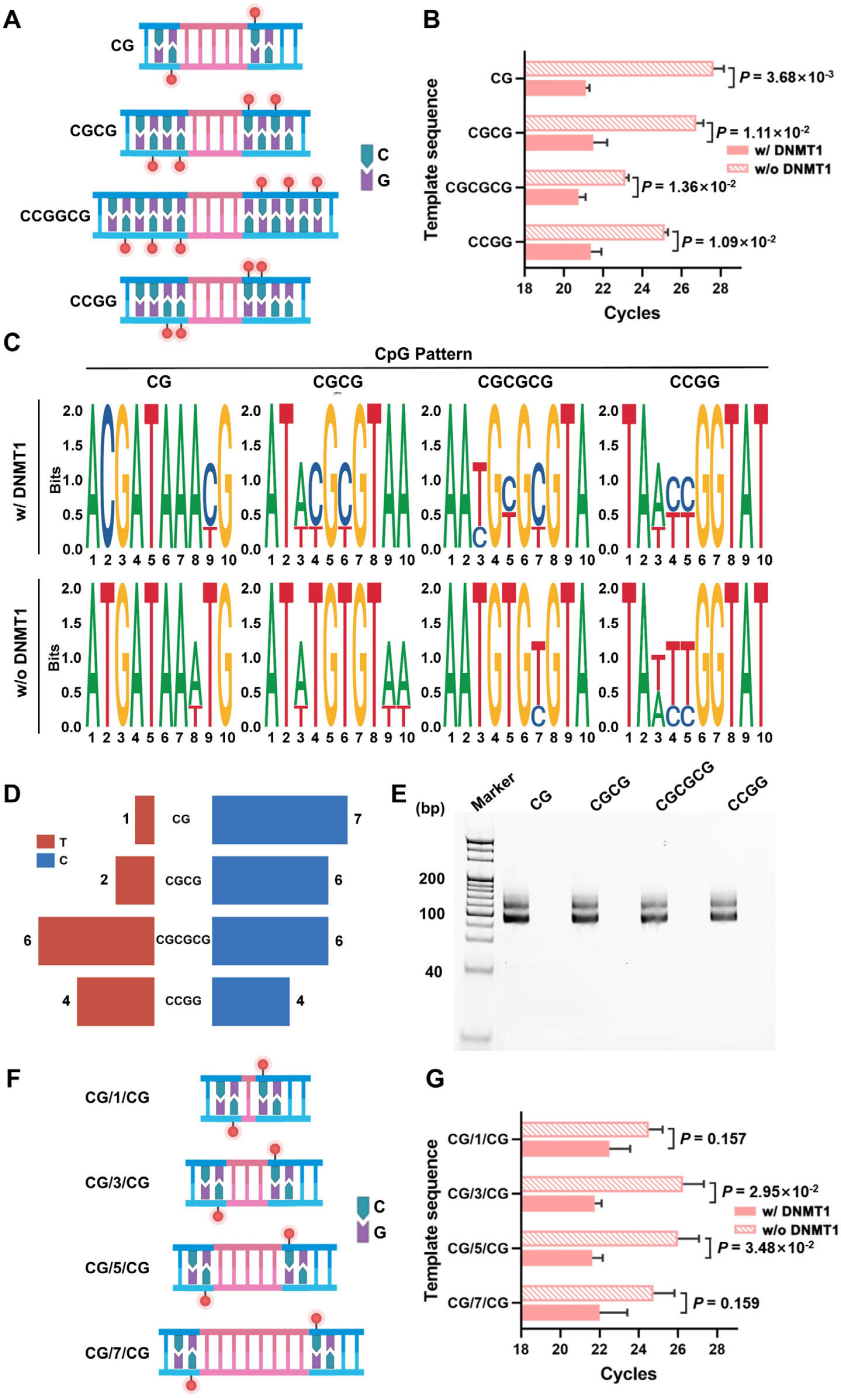
Optimization of the experimental conditions of DIVA. A) CG pattern plots of four staggered hemimethylated structures. B) The qPCR results for different GC patterns in the presence (w/) of DNMT1 or the absence (w/o) of DNMT1, *C*_DNA substrate_ = 250 fm, *C*_primer_ = 10 µm, *C*_DNMT1_ = 2×10^−3^ U mL^−1^, data presented: Mean ± SD. C) TA clone sequencing for the cytosine to thymine changes in the presence (w/) of DNMT1 or the absence (w/o) of DNMT1, *C*_DNA substrate_ = 250 nm, *C*_DNMT1_ = 2×10^−3^ U mL^−1^. D) Butterfly chart for the transition efficiency of the four CpG patterns. E) SDS‐PAGE of four GC patterns with or without DNMT1. F) CG pattern plots of the length of four inter‐CpG spacer sequences G) The qPCR for different length of inter‐CpG spacer sequences in the presence (w/) of DNMT1 or the absence (w/o) of DNMT1, *C*_DNA substrate_ = 250 fm, *C*_primer_ = 10 µm, *C*_DNMT1_ = 2×10^−3^ U mL^−1^, data presented: Mean ± SD.

Since different CpG patterns were crucial for the efficiency of DNA methyltransferase, we focused on optimizing the sequence design. We first optimized the reaction time, buffer types, and substrate concentrations (Figure S3A–D, Supporting Information). Under these optimal reaction conditions, four CpG patterns, including ‐CG‐, ‐CGCG‐, ‐CGCGCG‐, and ‐CCGG‐ were designed and evaluated for DNMT1 detection using DIVA (Table S2, Supporting Information). For each CG pattern, we designed a staggered hemimethylated structure for evaluation (**Figure** **2**A), using substrate and DNMT1 concentrations of 250 fm and 2×10^−3^ U mL^−1^, respectively. The qPCR results indicated that the ‐CG‐ pattern was optimal, exhibiting the most effective discrimination between positive samples (21.12 ± 0.13) and the control (28.03 ± 0.65) with a *p*‐value of 3.68 ×10^−3^ (Figure 2B). The tandem CpG pattern indicated less effective, while triplet CpG or CCGG showed the worst discrimination ability for DNMT1 detection using DIVA.

We also performed TA‐clone sequencing to demonstrate the successful transition from cytosine to thymine (Figure 2C). In the presence of DNMT1, methylated cytosines could not be deaminated, and thus remained as cytosine during sequencing. Since the bisulfite conversion efficiency was extremely high, the cytosine – to – thymine ratio represented the methylated efficiency of DNMT1. For the ‐CG‐ pattern, DNMT1 methylated most cytosines, preventing their conversion to thymine. The other three patterns showed obviously less efficient methylation. However, without DNMT1, all cytosines were converted to thymine due to bisulfite's high efficiency.^[^ ^36^, ^37^ ^]^ The transition efficiency determined by TA clone sequencing was consistent with the qPCR results. As shown in Figure 2D, the ‐CG‐ and ‐CGCG‐ patterns exhibited the highest methylating efficiencies of 87.5% and 75%, respectively, while the other two were less efficient. The PAGE gel showed clear differences in patterns with and without DNMT1 (Figure 2E). Considering the high transition efficiency of bisulfite conversion, we concluded that the methylation process was the rate‐determining step in DIVA. We then investigated how the length of the inter‐CpG spacer sequences affected DIVA efficiency (Figure 2F). Four substrates with different inter‐CpG spacer lengths were designed. The spacer length strongly influenced transition efficiency, with optimal differentiation achieved when 5 base pairs separated two CpGs (Figure 2G).

Interestingly, the preferred CpG pattern may vary for different methyltransferases. M.SssI, the DNA methylase from *Spiroplasma sp*. strain MQ1,^[^ ^38^ ^]^ required different reaction condition. We optimized its nucleic acid sequence design, reaction time, buffer type, and substrate concentration (Table S3, Figure S4A–C, Supporting Information), and tested for the optimal sequence. The results indicated that the tandem ‐CGCG‐ provided the most efficient distinguishing ability (Figure S4D, Supporting Information).

### 2.3 In Vitro Detection of DNMT1 Activity

**Figure 3:**
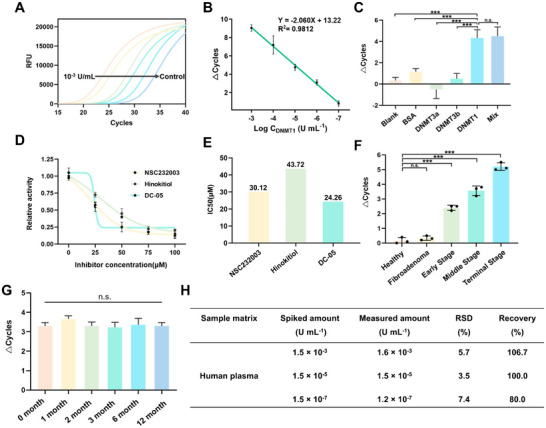
In vitro detection of DNMT1 activity. A) The in vitro detection range of DIVA. B) Linear regression curve for detection of DNMT1 with DIVA. *C* represented activity of DNMT1, *Δ cycle* represented the cycle difference to the negative control in the PCR process (n = 3), data presented: Mean ± SD. C) The specificity assay of different substances by DIVA, *C*_BSA_ = 1mg mL^−1^ *, C*_DNMT3A_ = 1.5×10^−3^ U mL^−1^, *C*_DNMT3B_ = 1.5×10^−3^ U mL^−1^, *C*_DNMT1_ = 1.5×10^−3^ U mL^−1^, data presented: Mean ± SD, *p* value: *** < 0.001, n.s., not significant. D) Relative activity curves of three DNMT1 inhibitors, data presented: Mean ± SD. E) The IC50 values of the inhibitors were determined by DIVA. F) The stability assay of three parallel testing on the random samples from healthy individual, patient with fibroadenoma, or early‐/middle‐/terminal‐stage breast cancer, data presented: Mean ± SD, *p* value: *** < 0.001, n.s., not significant. G) The stability assay tested at 0, 1, 2, 3, 6, and 12 months by DIVA, data presented: Mean ± SD, *p* value: n.s., not significant. H) The spiking recovery of the quantitative detection of DNMT1.

The optimized substrate and reaction conditions were used to test the quantification ability of this reaction. Commercially available DNMT1 was used for the detection of DIVA in vitro. Compared to the negative control, 10^−7^ U mL^−1^ DNMT1 showed significant differences (**Figure** **3**A). The active concentration of DNMT1 maintained an excellent linear relationship with the Δ cycle over the range of 10^−3^ – 10^−7^ U mL^−1^, with the regression equation Δ*Cycle*  =   − 2.060lg*C* + 13.22 (*R* ^2^ = 0.9812) (Figure 3B). We further tested DIVA's specificity and anti‐interference capability. Using two common *de novo* methyltransferases in human, DNMT3A and DNMT3B, along with BSA as interfering substances (Figure 3C), we found that these three substances did not significantly affect the reaction outcome. Moreover, even a mixture of these substances showed no significant interference in DNMT1 detection.

Additionally, we tested three commonly used DNMT1 inhibitors, NSC232003, hinokitiol, and DC_05, and determined their IC50 values using DIVA (Figure 3D). DC_05 exhibited the highest inhibiting efficiency because it directly inhibited the interactions between DNMT1 and DNA.^[^ ^39^ ^]^ The results showed the values of 30.12, 43.72, and 24.26 µm, respectively, which were consistent with literature values (Figure 3E). DIVA exhibited excellent stability in parallel sample testing (Figure 3F), and for the test of same sample at 0, 1, 2, 3, 6, and 12 months post‐preparation, no significant difference was found, indicating the assay's outstanding long‐term performance (Figure 3G).

Furthermore, we used human plasma as a simulated sample, performing quantitative detection of DNMT1 through a spiking recovery approach at high (1.5 × 10^−3^ U mL^−1^), medium (1.5 × 10^−5^ U mL^−1^), and low (1.5 × 10^−7^ U mL^−1^) concentrations (Figure 3H). Three parallel tests confirmed DIVA's good detection efficiency and stability in these samples. However, given that healthy human plasma contains baseline DNMT1, the recovery efficiency at low concentrations was slightly affected.

Similarly, when we applied this method to detect methyltransferase M.SssI, the results revealed comparable detection limit (10^−7^ U mL^−1^) and a similar linear detection range (10^−3^ – 10^−7^ U mL^−1^) (Figure S5A,B, Supporting Information). We assessed the stability and interference resistance for M.SssI detection using the same approach, and the results yielded consistency with DNMT1 (Figure S5C,D, Supporting Information), demonstrating the method's overall robustness.

### 2.4 Human Plasma Detection and Tumor Burden

**Figure 4:**
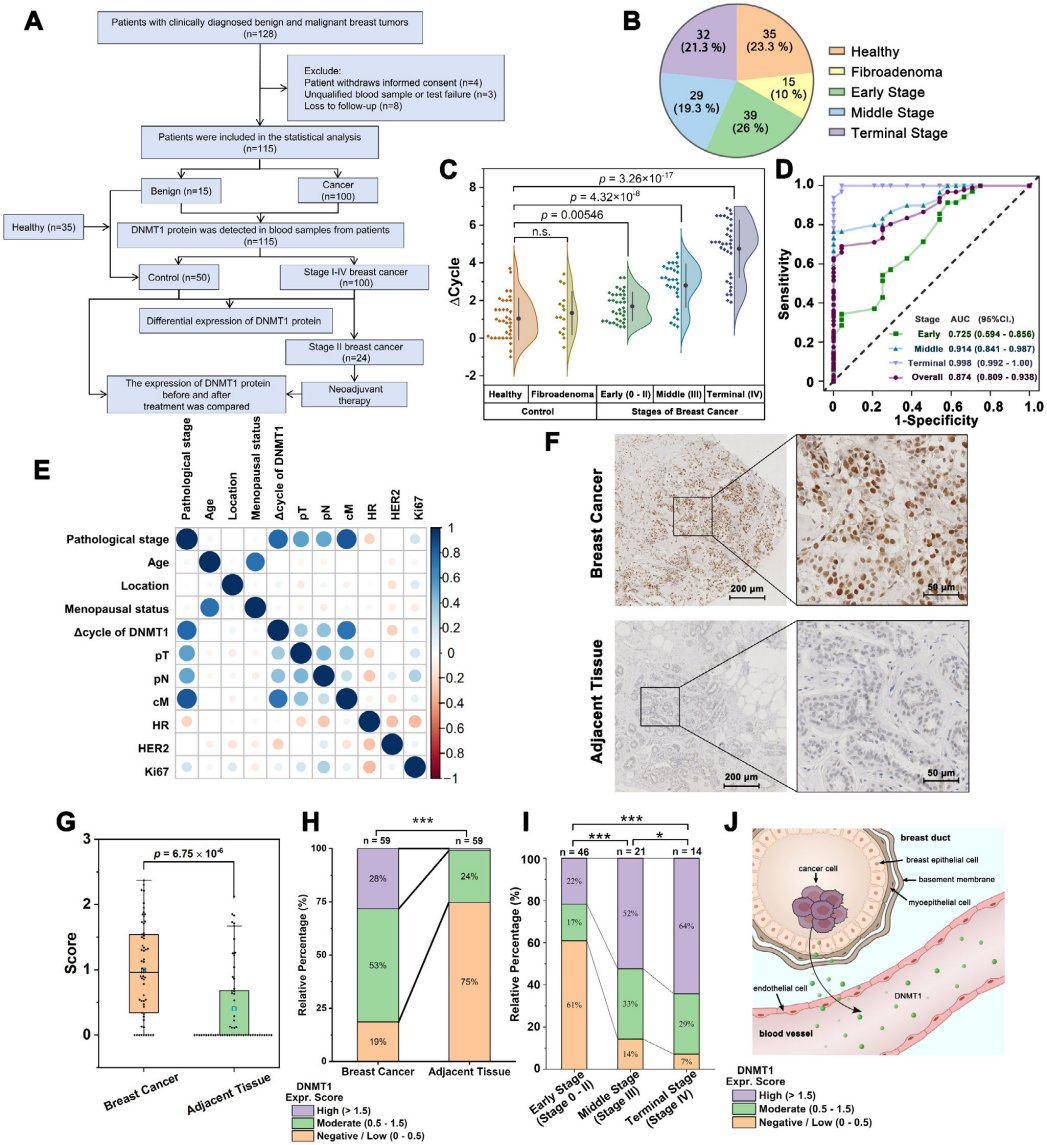
Plasma DNMT1 detection and exploration of the origin of DNMT1. A) The flow chart for the recruitment and exclusion of samples in this study. B) The number and proportion of samples recruited in this study. C) The distribution of DIVA results among the groups with different tumor burden, data presented: Mean ± SD. D) The ROC curve of DIVA on the different stages breast cancer patients. Early: AUC = 0.725, 95%CI. = 0.594 to 0.856, *p* = 0.0035, n = 35; Middle: AUC = 0.914, 95%CI. = 0.841 to 0.987, *p* < 0.0001, n = 30; Terminal: AUC = 0.998, 95%CI. = 0.992 to 1.00, *p* < 0.0001, n = 32; Overall: AUC = 0.874, 95%CI. = 0.809 to 0.938, *p* < 0.0001, n = 97. E) Correlation matrix between Δ cycle of DNMT1 and various clinical features. The size of the circles represents the strength of the correlation, red indicating a positive correlation and blue indicating a negative correlation. F) The IHC for the DNMT1 of breast cancer tissue and the adjacent tissue from the same individual. G) The IHC score for DNMT1 among breast cancer tissues and the paired adjacent tissues, upper whisker: Q3 + 1.5×IQR, lower whisker: Q1 – 1.5×IQR, box: median ± IQR, square: mean. H) The proportion of IHC scores for DNMT1 among breast cancer tissues and the paired adjacent tissues, examined by Chi‐square test, *p* value: *** < 0.001. I) The proportion of IHC scores for DNMT1 among three tumor stages of breast cancer, examined by Chi‐square test, *p* value: * <0.05, *** < 0.001. J) Possible mechanism of DNMT1 secretion in breast cancer cells.

Due to the upstream role of epigenetic alterations in breast cancer phenotypes, we attempted to establish a method for tumor burden assessment by monitoring the DNMT1 activity levels in plasma samples from patients with different stages of breast cancer. A total of 100 patients, who were diagnosed with stage I to IV breast cancer at the Department of Breast and Thyroid Surgery, Southwest Hospital, were enrolled in this prospective clinical‐test cohort study (**Figure** **4**A). This study was approved by the Clinical Research Ethics Committee of The First Affiliated Hospital of the Army Medical University, Chongqing, China. Written informed consent was obtained, and patients were compensated for participation. According to the clinical practice guidelines for breast cancer from National Comprehensive Cancer Network (NCCN),^[^ ^40^ ^]^ we divided the evaluable patients into three tumor stages based on their TNM stages: early sage (stage I–II, n = 39, 26%), medium stage (stage III, n = 29, 19.3%), and terminal (stage IV, n = 32, 21.3%). Additionally, fibroadenoma patients (n = 15, 10.0%) and healthy individuals (n = 35, 23.3%) were enrolled as negative controls (Figure 4B). The baseline information of the 100 breast cancer patients is presented in **Table** **1**.

**Table 1:** Baseline characteristics of breast cancer patients in different pathological stages.

Characteristics	I–II^a)^ (n = 39)	III^a)^ (n = 29)	IV^a)^ (n = 32)
Age [mean (SD)], years	48.00 [9.52]	50.14 [10.17]	49.66 [8.23]
Location of the tumor, n (%)			
Left breast	20 (51.28)	10 (34.48)	17 (53.12)
Right breast	19 (48.72)	19 (65.52)	15 (46.88)
Menopausal status, n (%)			
Premenopausal	14 (35.90)	10 (34.48)	8 (25.00)
Postmenopausal	25 (64.10)	19 (65.52)	24 (75.00)
T stage, n (%)			
x	0	0	9 (28.12)
1	8 (20.51)	0	1 (3.12)
2	31 (79.49)	19 (65.52)	10 (31.25)
3	0	6 (20.69)	3 (9.38)
4	0	6 (20.69)	3 (9.38)
N stage, n (%)			
0	16 (41.03)	1 (3.45)	11 (34.38)
1	23 (58.97)	4 (13.79)	6 (18.75)
2	0	17 (58.62)	3 (9.38)
3	0	7 (24.14)	12 (37.50)
M stage, n (%)			
0	39 (100.00)	29 (100)	0
1	0	0	32 (100)
HR status, n (%)			
Negative	10 (25.64)	17 (58.62)	16 (50.00)
Positive	29 (74.36)	12 (41.38)	16 (50.00)
HER2 status, n (%)			
0, 1+, 2+ and FISH‐	22 (56.41)	8 (27.59)	21 (65.62)
3+, 2+ and FISH+	17 (43.59)	21 (72.41)	11 (34.38)
Ki67 [median (IQR)]	20.00 (10.00, 40.00)	30.00 (15.00, 50.00)	30.00 (20.00, 40.00)

^a)^ The Pathological TNM stage.

Abbreviations: HR, hormone status; HER2, human epidermal growth factor receptor 2; FISH, fluorescence in situ hybridization; IQR, interquartile range.

We examined the DNMT1 activity in plasma using DIVA across the 150 samples. We found that the DNMT1 activity (determined as the ΔCT value of qPCR) exhibited a significant correlation with tumor stage (Figure 4C). As the tumor stage increased, a pronounced and significant elevation in DNMT1 activity was revealed. The healthy group had the lowest DNMT1 activity in plasma (ΔCT, 1.32; IQR, −0.02 – 1.78), and the fibroadenoma group showed no significant difference compared to the healthy group (ΔCT, 1.44; IQR, 0.05 – 1.82). The DNMT1 activity exhibited a noticeable increase in the early‐stage group (ΔCT, 1.61; IQR, 1.07 – 2.35, *P* = 0.00546), with further elevation in the medium‐stage group (ΔCT, 2.92; IQR, 1.57 – 3.86, *p* < 0.0001), and reaching its peak in the terminal‐stage group (ΔCT, 3.76; IQR, 3.13 – 5.81, *p* < 0.0001). Additionally, we collected samples from six patients with mastitis, and found no significant difference when compared to the healthy individuals (Figure S6, Supporting Information).

To validate the robustness of DIVA in assessing tumor burden in breast cancer patients, we re‐recruited 97 patients at various stages, as well as 24 healthy participants. We employed DIVA to determine DNMT1 activity in their peripheral blood and compared it with the biopsy‐confirmed outcomes. The receiver‐operating characteristic (ROC) analysis is shown in Figure 4D. The area under the curve (AUC) for overall performance was 0.874 (95%CI. = 0.809 to 0.938, *p* < 0.0001, n = 97), indicating excellent predictive ability. In the ROC analyses across different stages, DIVA demonstrated a high predictive capability, and its reliability gradually increased along tumor progression. especially for the prediction of the terminal stage (AUC = 0.998, 95%CI. = 0.992 to 1.00, *p* < 0.0001, n = 32).

The correlation matrix demonstrated a significant relationship between DNMT1 activity and both the pathological staging and TNM status of breast cancer patients (Figure 4E). Notably, we observed a strong association between DNMT1 levels and metastasis, suggesting that DNMT1 is more likely to be released into the peripheral blood during tumor metastasis. Furthermore, we found no significant correlation between DNMT1 activity and patient age, tumor location, menopausal status, or ER, HER2, and Ki67 expression. These results suggested that the DNMT1 activity in plasma could serve as a promising biomarker for tumor burden assessment in breast cancer.

### 2.5 Exploration of the Origin of DNMT1

To further elucidate the mechanisms behind increased DNMT1 activity in plasma and its potential sources, we analyzed tissue microarray of 59 patients and evaluated the DNMT1 expression intensity in cancer regions and paired adjacent tissue using immunohistochemistry (IHC). The IHC results showed that the nucleus of cancer cells overexpressed DNMT1 while adjacent tissue cells showed significant lower expression (Figure 4F). We used the IHC score to evaluate DNMT1 expression in the specimens, as assessed by two independent experienced pathologists (see details in Materials and Methods). We also observed an obvious and significant overexpression of DNMT1 in breast cancer tissue (score, 0.96; IQR, 0.34 – 1.54; *p* < 0.0001) compared to the adjacent tissues (Figure 4G).

We further categorized the score values into three groups (high, > 1.5; moderate, 0.5 – 1.5; low, < 0.5), revealing a statistically significant difference between breast cancer (low, 18.57%; moderate, 53.11%; high, 28.32%) and adjacent tissue (low, 74.76%; moderate, 24.29%; high, 0.94%) (Figure 4H). The high expression of DNMT1 in tumor cells was previously linked to the binding of MYC to the DNMT1 promoter region and subsequent activation of its transcription,^[^ ^41^ ^]^ where DNMT1 can be further recruited by enhancer of EZH2 to methylate the promoter sequence of tumor suppressors.^[^ ^42^ ^]^

When we correlated the scoring outcomes tumor burden levels, we observed that higher burden corresponded to an increased proportion of high scores (early‐stage, 22%; middle‐stage, 52%; terminal‐stage, 64%) and decreased proportion of low scores (early‐stage, 61%; middle‐stage, 14%; terminal‐stage, 7%) (Figure 4I). The observed expression of DNMT1 in cancer tissues increased progressively with disease stage progression, consistent with previous reports showing that DNMT1 expression correlated with high stage breast cancers.^[^ ^43^, ^44^ ^]^ This patten also aligned with DNMT1 activity levels in patient plasma.

We inferred the following mechanism: 1) Proliferating cancer cells in the breast duct secret DNMT1; 2) The secreted DNMT1 passes through breast duct and enters the circulating system; 3) Higher tumor burden results in increased DNMT1 secretion, which can then be detected in plasma (Figure 4J). Two lines of evidence support this mechanism. First, we observed DNMT1 staining in the cytoplasm, suggesting possible transportation of DNMT1 from the nucleus (Figure S7, Supporting Information); Second, we detected active DNMT1 in extracellular vehicles (EVs) isolated from four different breast cancer cells, indicating that circulating DNMT1 can be excreted via EVs. (Figure S8, Supporting Information). However, further experiments are needed to confirm the exact pathways of DNMT1 transportation into the cytoplasm and its functionality in peripheral blood.

### 2.6 Monitoring the Efficacy of Neoadjuvant Therapy for Breast Cancer using DNMT1 Activity

Given that DNMT1 activity in plasma increased with breast cancer stage progression due to overexpression during cancer cell proliferation, we hypothesized that DNMT1 activity would decrease following successful treatment and serve as a marker for monitoring therapeutic efficacy.^[^ ^45^ ^]^ To eliminate potential surgical effects on plasma DNMT1 activity, we enrolled 22 patients with operable stage II invasive ductal carcinoma who were receiving ECPy‐THPy (epirubicin, cyclophosphamide and pyrotinib followed by docetaxel, trastuzumab, and pyrotinib), TcbHPy (docetaxel, carboplatin, trastuzumab, and pyrotinib) or TAC (docetaxel, epirubicin and cyclophosphamide) neoadjuvant therapy. We monitored all patients using both ultrasound image and plasma DNMT1 detection during their treatment course. We collected peripheral blood samples at two timepoints: pre‐treatment and post‐treatment (pre‐surgery), to monitor changes in DNMT1 activity. After completing neoadjuvant therapy, we classified patients into four groups according to treatment response based on the RECIST (Response Evaluation Criteria in Solid Tumors) guidelines:^[^ ^46^ ^]^ complete response (CR), partial response (PR), stable disease (SD), and progressive disease (PD). We then compared changes in DNMT1 activity among these groups. The patients’ baseline characteristic indicated no significant enrollment bias (Table S4, Supporting Information).

**Figure 5:**
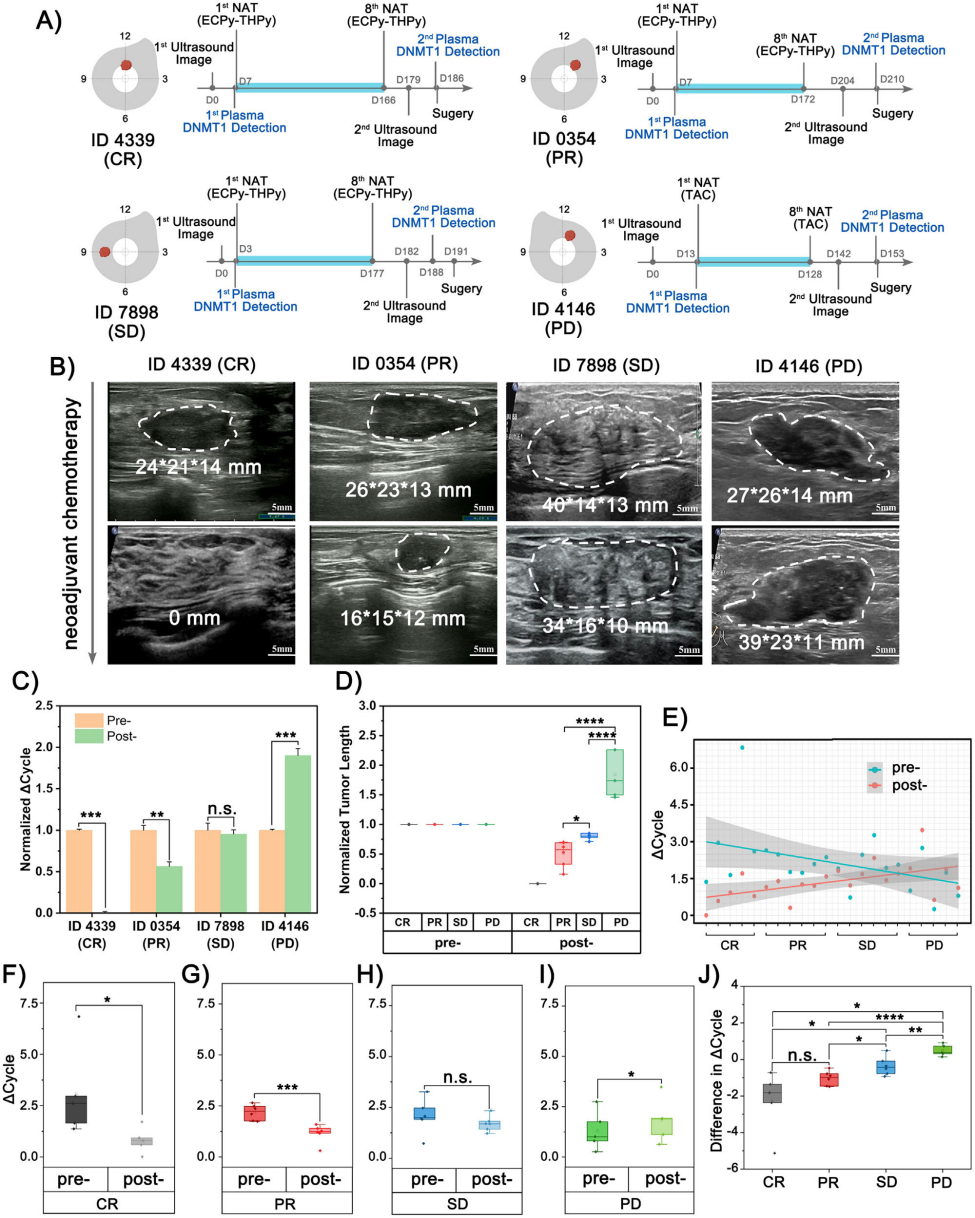
Monitoring the efficacy of neoadjuvant therapy for breast cancer using DNMT1 activity. A) The timeline of the therapeutic regimens, ultrasound image and DNMT1 detections. B) The ultrasound images before and after neoadjuvant therapies. C) The qPCR results for detection DNMT1 of neoadjuvant therapies patients by DIVA, data presented: Mean ± SD, *p* value: ** <0.01, *** < 0.001, n.s., not significant. D) Changes in tumor length before and after neoadjuvant therapies by ultrasound images, upper whisker: Q3 + 1.5×IQR, lower whisker: Q1 – 1.5×IQR, box: median ± IQR, square: mean, *p* value: * <0.05, **** < 0.0001. E) Trend analysis of DNMT1 activity by DIVA in different efficacy groups. F) The DNMT1 activity of the CR group before and after neoadjuvant therapies, upper whisker: Q3 + 1.5×IQR, lower whisker: Q1 – 1.5×IQR, box: median ± IQR, square: mean, *p* value: * <0.05. G) The DNMT1 activity of the PR group before and after neoadjuvant therapies, upper whisker: Q3 + 1.5×IQR, lower whisker: Q1 – 1.5×IQR, box: median ± IQR, square: mean, *p* value: *** <0.001. H) The DNMT1 activity of the SD group before and after neoadjuvant therapies, upper whisker: Q3 + 1.5×IQR, lower whisker: Q1 – 1.5×IQR, box: median ± IQR, square: mean, n.s., not significant. I) The DNMT1 activity of the PD group before and after neoadjuvant therapies, upper whisker: Q3 + 1.5×IQR, lower whisker: Q1 – 1.5×IQR, box: median ± IQR, square: mean, *p* value: * <0.05. J) The differences in DNMT1 levels among the groups before and after neoadjuvant therapy, upper whisker: Q3 + 1.5×IQR, lower whisker: Q1 – 1.5×IQR, box: median ± IQR, square: mean, *p* value: * <0.05, ** <0.01, *** < 0.0001, n.s., not significant.

We first validated the consistency between monitoring DNMT1 activities and treatment outcomes using four representative patients with different prognoses as case examples (patient ID 4339, CR; ID 0354, PR; ID 7898, SD; ID 4146, PD). We monitored, recorded, and evaluated the timeline of therapeutic regimens, ultrasound image, and DNMT1 detection (**Figure** **5**A). The ultrasound images clearly demonstrated the effectiveness of neoadjuvant therapies (Figure 5B). In ID 4339 patient, the tumor tissue disappeared in the image; in ID 0354 patient, the tumor length decreased to 61% of its original length; for the SD and PD patients, the tumor lengths were 85% and 144% of their original lengths, respectively.

Our DIVA method revealed the same trend of changes when measuring plasma DNMT1 activities (Figure 5C). In CR and PR patients, we observed significantly reduced DNMT1 activities after neoadjuvant therapies. However, in SD and PD patients, DNMT1 activities remained similar or increased 1.9‐fold after treatment. We expand our analysis to 22 patients in total to evaluate the consistency between DNMT1 activity changes and neoadjuvant therapy response (Figures S9–S16, Supporting Information). Following the RECIST criteria, we compared changes in tumor length measured by ultrasound images before and after neoadjuvant therapies, then grouped patients according to treatment outcomes (Figure 5D).

The overall results indicated more significant decreases in DNMT1 activities among CR and PR patients following treatment, while SD and PD patients showed minimal changes (Figure 5E). Specifically, in the CR group, mean DNMT1 activity decreased from 2.61(IQR, 1.65 – 2.96) to 0.79 (IQR, 0.59 – 0.93) (Figure 5F), which was comparable to the value of healthy individuals. In the PR group, it declined from 2.24 (IQR, 1.77 – 2.48) to 1.24 (IQR, 1.16 – 1.40) (Figure 5G). We found no significant difference in the SD group (Figure 5H) and observed a clear elevation of DNMT1 activity in the PD group from 1.01 (IQR, 0.80 – 1.75) to 1.89 (IQR, 1.12 – 1.92) (Figure 5I).

The magnitude of DNMT1 activity changes correlated with treatment outcome, with patients showing better treatment results demonstrating more notable decreases in DNMT1 activities. Moreover, the extent of DNMT1 changes differed significantly among patients with different outcomes (Figure 5J). These findings suggest that changes in plasma DNMT1 activity could serve as an effective biomarker for predicting neoadjuvant therapy effectiveness in breast cancer patients.

## 3 Conclusion

DNMT1, which restores specific methylation patterns to hemimethylated strands during replication, plays a crucial role in tumor cell growth, migration, and invasion. Importantly, DNMT1‐mediated changes in genomic methylation precede tumor cell phenotypic changes temporally. Therefore, we developed DIVA, a sensitive method for DNMT1 detection. The DIVA system comprises three inter‐linked modules including DNA methylation, cytosine deamination, and truncation.

We utilized varying levels of DNMT1 activity to create methylation differences, thereby generating both methylated and unmethylated DNA sequences within the substrate. Next, we subjected the unmethylated sites to bisulfite conversion, converting them into uracil through deamination. Finally, we employed a combination of *afu* UDG and endo IV to cleave at uracil positions. As different DNMT1 activity levels directly affected the extent of substrate cleavage, we accurately detected DNMT1 levels as low as 10^−7^ U mL^−1^ through RT‐PCR, requiring minimal time (≈6 h) and cost (≈1.5 USD per sample), which demonstrated a superior performance in the aspects of sensitivity, cost‐effectiveness and clinical utility.

150 clinical samples were examined with DIVA, confirming the robustness of DNMT1 in assessing tumor burden in breast cancer patients. Another 121 clinical samples were recruited and revealed significant differences in plasma DNMT1 activity among patients with different stages of breast cancer. Moreover, plasma DNMT1 levels increased progressively with advancing breast cancer stage. These findings aligned with our IHC analysis of cancer tissues. Based on these results, we hypothesize that plasma DNMT1 originates from tumor tissues, although further research is needed to confirm this hypothesis. Finally, we applied this method to monitor therapeutic efficacy in 22 patients undergoing neoadjuvant treatment for breast cancer. Our results indicated that patients responding well to neoadjuvant treatment showed significant decreases in DNMT1 levels following treatment, while patients with suboptimal responses showed no significant changes or even increased DNMT1 levels.

In subsequent studies, we will focus on a more in‐depth investigation into the secreting pathway of DNMT1. Meanwhile, we intend to clarify whether the plasma DNMT1 could influence tumor progression and metastasis through additional methylation of the nucleic acids in peripheral blood. We believe that the development of DIVA represents an important step toward establishing a meaningful early diagnostic test for breast cancer, and that plasma DNMT1 activity shows promise as a biomarker for both tumor burden assessment and treatment monitoring in clinical perspective.

## 4 Experimental Section

### Human Ethics Statement

This study was approved by the Clinical Research Ethics Committee of The First Affiliated Hospital of the Army Medical University, Chongqing, China (Approval number: AIIT2023060).

### Statistical Analysis

For column chart, data are presented as means ± SD of at least ≥3 independent measurements. For box plot, data are presented as median ± IQR, upper whisker indicates Q3 + 1.5×IQR, lower whisker indicates Q1 – 1.5×IQR. Statistical analysis was performed by linear regression with 95% confidence intervals (CI), two‐tailed Student's *t* test, or one‐way analysis of variance (ANOVA). The level of statistical significance was indicated on the graphs using asterisks (*, **, *** or ****) for *p* values less than 0.05, 0.01, 0.001 or 0.0001, respectively. Data analysis was performed with GraphPad Prism 8.0 (GraphPad Software, USA) and Microsoft Excel 2019 (version 2412).

## Conflict of Interest

The authors declare no conflict of interest.

## Author Contributions

Y.W., G.Z. and Z.Z. contributed equally to this work as co‐authors. M.G. and X.Q. performed conceptualization; Y.W, G.Z., and Z.Z. performed methodology; M.Z., J.C., K.W., and L.L. performed investigation; M.G. and Y.W. wrote the original draft; J.B. and M.C. reviewed and edited the final manuscript; M.G. and X.Q. performed funding acquisition; M.G. and X.Q. acquired resources; M.G. and X.Q performed supervision.

## Supporting information



Supporting Information

## Data Availability

The data that support the findings of this study are available from the corresponding author upon reasonable request.

## References

[1] R. L. Siegel , T. B. Kratzer , A. N. Giaquinto , H. Sung , A. Jemal , Ca‐Cancer J. Clin. 2025, 75, 10. 39817679 10.3322/caac.21871 PMC11745215

[2] A. N. Giaquinto , H. Sung , L. A. Newman , R. A. Freedman , R. A. Smith , J. Star , A. Jemal , R. L. Siegel , Ca‐Cancer J. Clin. 2024, 74, 477. 39352042 10.3322/caac.21863

[3] U. Anand , A. Dey , A. K. S. Chandel , R. Sanyal , A. Mishra , D. K. Pandey , V. De Falco , A. Upadhyay , R. Kandimalla , A. Chaudhary , J. K. Dhanjal , S. Dewanjee , J. Vallamkondu , J. M. Pérez de la Lastra , Genes Dis. 2023, 10, 1367. 37397557 10.1016/j.gendis.2022.02.007 PMC10310991

[4] I. Meattini , C. Becherini , L. Boersma , O. Kaidar‐Person , G. N. Marta , A. Montero , B. V. Offersen , M. C. Aznar , C. Belka , A. M. Brunt , S. Dicuonzo , P. Franco , M. Krause , M. MacKenzie , T. Marinko , L. Marrazzo , I. Ratosa , A. Scholten , E. Senkus , H. Stobart , P. Poortmans , C. E. Coles , Lancet Oncol. 2022, 23, 21. 34973228 10.1016/S1470-2045(21)00539-8

[5] Y. Zou , J. Xie , S. Zheng , W. Liu , Y. Tang , W. Tian , X. Deng , L. Wu , Y. Zhang , C.‐W. Wong , D. Tan , Q. Liu , X. Xie , Int. J. Surg. 2022, 107, 106936. 36341760 10.1016/j.ijsu.2022.106936

[6] Y. Li , H. Zhang , Y. Merkher , L. Chen , N. Liu , S. Leonov , Y. Chen , J. Hematol. Oncol. 2022, 15, 121. 36038913 10.1186/s13045-022-01341-0 PMC9422136

[7] C. Yau , M. Osdoit , M. Van Der Noordaa , S. Shad , J. Wei , D. De Croze , A.‐S. Hamy , M. Laé , F. Reyal , G. S. Sonke , T. G. Steenbruggen , M. Van Seijen , J. Wesseling , M. Martín , M. Del Monte‐Millán , S. López‐Tarruella , J. C. Boughey , M. P. Goetz , T. Hoskin , R. Gould , V. Valero , S. B. Edge , J. E. Abraham , J. M. S. Bartlett , C. Caldas , J. Dunn , H. Earl , L. Hayward , L. Hiller , E. Provenzano , et al., Lancet Oncol. 2022, 23, 149.34902335

[8] M. J. M. Magbanua , L. B. Swigart , H.‐T. Wu , G. L. Hirst , C. Yau , D. M. Wolf , A. Tin , R. Salari , S. Shchegrova , H. Pawar , A. L. Delson , A. DeMichele , M. C. Liu , A. J. Chien , D. Tripathy , S. Asare , C.‐H. J. Lin , P. Billings , A. Aleshin , H. Sethi , M. Louie , B. Zimmermann , L. J. Esserman , L. J. V. t. Veer , Ann. Oncol. 2021, 32, 229. 33232761 10.1016/j.annonc.2020.11.007 PMC9348585

[9] M. J. M. Magbanua , L. Brown Swigart , Z. Ahmed , R. W. Sayaman , D. Renner , E. Kalashnikova , G. L. Hirst , C. Yau , D. M. Wolf , W. Li , A. L. Delson , S. Asare , M. C. Liu , K. Albain , A. J. Chien , A. Forero‐Torres , C. Isaacs , R. Nanda , D. Tripathy , A. Rodriguez , H. Sethi , A. Aleshin , M. Rabinowitz , J. Perlmutter , W. F. Symmans , D. Yee , N. M. Hylton , L. J. Esserman , A. M. DeMichele , H. S. Rugo , et al., Cancer Cell 2023, 41, 1091. 37146605 10.1016/j.ccell.2023.04.008 PMC10330514

[10] Y. Zhu , W. Li , F. Lan , S. Chen , X. Chen , X. Zhang , X. Yan , Y. Zhang , Interdiscip. Med. 2024, 2, 20230043.

[11] E. Nolan , G. J. Lindeman , J. E. Visvader , Cell 2023, 186, 1708. 36931265 10.1016/j.cell.2023.01.040

[12] K. K. Wong , Semin. Cancer Biol. 2021, 72, 198. 32461152 10.1016/j.semcancer.2020.05.010

[13] W. Zhang , J. Xu , Biomark Res. 2017, 5, 1. 28127428 10.1186/s40364-017-0081-z PMC5251331

[14] V. Davalos , M. Esteller , Ca‐Cancer J. Clin. 2023, 73, 376. 36512337 10.3322/caac.21765

[15] L. R. Varga , T. Marić , A. K. Bojanac , K. Horvatović , M. Popovic , A. Kulić , T. Silovski , M. Sirotkovic‐Skerlev , M. Krizic , K. Bulić , H. Silovski , N. D. Plavetic , Ann. Oncol. 2023, 34, S304.

[16] Z. Chen , Y. Zhang , Annu Rev. Biochem. 2020, 89, 135. 31815535 10.1146/annurev-biochem-103019-102815

[17] B. Jin , J. Ernst , R. L. Tiedemann , H. Xu , S. Sureshchandra , M. Kellis , S. Dalton , C. Liu , J.‐H. Choi , K. D. Robertson , Cell Rep. 2012, 2, 1411. 23177624 10.1016/j.celrep.2012.10.017 PMC3625945

[18] B. Jin , K. D. Robertson , in Epigenetic Alterations in Oncogenesis (Ed.: A. R. Karpf ), Vol. 754, Springer, New York, NY 2013, pp. 3–29.

[19] P. Mehdipour , R. Chen , D. D. De Carvalho , Nat. Cancer 2021, 2, 1000. 35121882 10.1038/s43018-021-00271-z

[20] P. Liu , F. Yang , L. Zhang , Y. Hu , B. Chen , J. Wang , L. Su , M. Wu , W. Chen , Front Pharmacol. 2022, 13, 958146. 36091786 10.3389/fphar.2022.958146 PMC9453300

[21] L. Yan , Nass, S. J. , Smith, D. , Nelson, W G. , Herman, J G. , N. E. Davidson , Cancer Biol. Therapy 2003, 2, 552. 10.4161/cbt.2.5.469 14614325

[22] X. Liu , C. Li , R. Zhang , W. Xiao , X. Niu , X. Ye , Z. Li , Y. Guo , J. Tan , Y. Li , Cellular Signalling 2018, 51, 243. 30121333 10.1016/j.cellsig.2018.08.011

[23] W. W. Du , W. Yang , X. Li , F. M. Awan , Z. Yang , L. Fang , J. Lyu , F. Li , C. Peng , S. N. Krylov , Y. Xie , Y. Zhang , C. He , N. Wu , C. Zhang , M. Sdiri , J. Dong , J. Ma , C. Gao , S. Hibberd , B. B. Yang , Oncogene 2018, 37, 5829. 29973691 10.1038/s41388-018-0369-y

[24] R. Pathania , S. Ramachandran , S. Elangovan , R. Padia , P. Yang , S. Cinghu , R. Veeranan‐Karmegam , P. Arjunan , J. P. Gnana‐Prakasam , F. Sadanand , L. Pei , C.‐S. Chang , J.‐H. Choi , H. Shi , S. Manicassamy , P. D. Prasad , S. Sharma , V. Ganapathy , R. Jothi , M. Thangaraju , Nat. Commun. 2015, 6, 6910. 25908435 10.1038/ncomms7910 PMC4410389

[25] H. Zhang , H. Dong , G. Yang , H. Chen , C. Cai , Anal. Chem. 2016, 88, 11108. 27730812 10.1021/acs.analchem.6b03163

[26] L. Song , T. Ma , F. Gong , L. He , Y. Wang , Q. Zhang , S. Zhang , Y. Wu , L. Liu , F. Yu , Sensor Actuat. B‐chem 2023, 385, 133610.

[27] S. Xiao , S. Guo , J. Han , Y. Sun , M. Wang , Y. Chen , X. Fang , F. Yang , Y. Mu , L. Zhang , Y. Ding , N. Zhang , H. Jiang , K. Chen , K. Zhao , C. Luo , S. Chen , Nucleic Acids Res. 2022, 50, 9. 10.1093/nar/gkab989 PMC8789064 34718755

[28] L. Fan , Y. Peng , B. Ning , H. Wei , Z. Gao , J. Bai , L. Guo , Anal. Chim Acta 2020, 1103, 164. 32081181 10.1016/j.aca.2019.12.058

[29] Q. Dai , C. Ye , I. Irkliyenko , Y. Wang , H.‐L. Sun , Y. Gao , Y. Liu , A. Beadell , J. Perea , A. Goel , C. He , Nat. Biotechnol. 2024, 42, 1559. 38168991 10.1038/s41587-023-02034-w PMC11217147

[30] L. Wang , M. Ren , Q. Zhang , B. Tang , C. Zhang , Anal. Chem. 2017, 89, 4488. 28306242 10.1021/acs.analchem.6b04673

[31] S. M. Daskalova , B. M. Eisenhauer , M. Gao , X. Feng , X. Ji , Q. Cheng , N. Fahmi , O. M. Khdour , S. Chen , S. M. Hecht , Bioorgan Med. Chem. 2020, 28, 115642. 10.1016/j.bmc.2020.115642 32773093

[32] C. Yang , Y. Yuan , M. Shen , K. Wang , H. Xu , Y. Wang , M. Chen , J. Bao , M. Gao , Anal. Chem. 2022, 94, 15887. 36325814 10.1021/acs.analchem.2c04010

[33] K. Jensen , R. Krusenstjerna‐Hafstrøm , J. Lohse , K. H. Petersen , H. Derand , Modern Pathol. 2017, 30, 180. 10.1038/modpathol.2016.176 27767098

[34] E. Shin , Y. Lee , J. S. Koo , J. Transl. Med. 2016, 14, 87. 27071379 10.1186/s12967-016-0840-x PMC4830007

[35] C. Wu , Y. Liu , W. Liu , T. Zou , S. Lu , C. Zhu , L. He , J. Chen , L. Fang , L. Zou , P. Wang , L. Fan , H. Wang , H. You , J. Chen , J. Fang , C. Jiang , Y. Shi , Adv. Sci. 2023, 10, 2202642. 10.1002/advs.202202642 PMC9811437 36382559

[36] M.‐B. W Ørntoft , S. Ø. Jensen , T. B. Hansen , J. B. Bramsen , C. L. Andersen , Epigenetics‐us 2017, 12, 626. 10.1080/15592294.2017.1334024 PMC5687322 28557629

[37] C. A. Leontiou , M. D. Hadjidaniel , P. Mina , P. Antoniou , M. Ioannides , P. C. Patsalis , PLoS One 2015, 10, 0135058. 10.1371/journal.pone.0135058 PMC4527772 26247357

[38] P. Renbaum , D. Abrahamove , A. Fainsod , G. G. Wilson , S. Rottem , A. Razin , Nucleic Acids Res. 1990, 18, 1145. 2181400 10.1093/nar/18.5.1145 PMC330428

[39] S. Chen , Y. Wang , W. Zhou , S. Li , J. Peng , Z. Shi , J. Hu , Y.‐C. Liu , H. Ding , Y. Lin , L. Li , S. Cheng , J. Liu , T. Lu , H. Jiang , B. Liu , M. Zheng , C. Luo , J. Med. Chem. 2014, 57, 9028. 25333769 10.1021/jm501134e

[40] W. J. Gradishar , M. S. Moran , J. Abraham , R. Aft , D. Agnese , K. H. Allison , B. Anderson , H. J. Burstein , H. Chew , C. Dang , A. D. Elias , S. H. Giordano , M. P. Goetz , L. J. Goldstein , S. A. Hurvitz , S. J. Isakoff , R. C. Jankowitz , S. H. Javid , J. Krishnamurthy , M. Leitch , J. Lyons , J. Mortimer , S. A. Patel , L. J. Pierce , L. H. Rosenberger , H. S. Rugo , A. Sitapati , K. L. Smith , M. L. Smith , H. Soliman , J. Natl. Compr. Canc. Ne 2022, 20, 691. 10.6004/jnccn.2022.0030 35714673

[41] S.‐Y. Wu , Y. Xiao , J.‐L. Wei , X.‐E. Xu , X. Jin , X. Hu , D.‐Q. Li , Y.‐Z. Jiang , Z.‐M. Shao , J. Immunother. Cancer 2021, 9, 002528. 10.1136/jitc-2021-002528 PMC8320259 34321275

[42] Y. Li , Q. He , X. Wen , X. Hong , X. Yang , X. Tang , P. Zhang , Y. Lei , Y. Sun , J. Zhang , Y. Wang , J. Ma , N. Liu , Cell Death Differ. 2019, 26, 1089. 30353102 10.1038/s41418-018-0208-2 PMC6748116

[43] Z. Yu , Q. Xiao , L. Zhao , J. Ren , X. Bai , M. Sun , H. Wu , X. Liu , Z. Song , Y. Yan , X. Mi , E. Wang , F. Jin , M. Wei , Mol. Carcinog. 2015, 54, 707. 24464625 10.1002/mc.22133

[44] R. Ben Gacem , M. Hachana , S. Ziadi , S. Ben Abdelkarim , S. Hidar , M. Trimeche , Hum. Pathol. 2012, 43, 1731. 22520950 10.1016/j.humpath.2011.12.022

[45] J. Yu , B. Qin , A. M. Moyer , S. Nowsheen , T. Liu , S. Qin , Y. Zhuang , D. Liu , S. W. Lu , K. R. Kalari , D. W. Visscher , J. A. Copland , S. A. McLaughlin , A. Moreno‐Aspitia , D. W. Northfelt , R. J. Gray , Z. Lou , V. J. Suman , R. Weinshilboum , J. C. Boughey , M. P. Goetz , L. Wang , J. Clin. Invest. 2018, 128, 2376. 29708513 10.1172/JCI97924 PMC5983332

[46] E. A. Eisenhauer , P. Therasse , J. Bogaerts , L. H. Schwartz , D. Sargent , R. Ford , J. Dancey , S. Arbuck , S. Gwyther , M. Mooney , L. Rubinstein , L. Shankar , L. Dodd , R. Kaplan , D. Lacombe , J. Verweij , Eur. J. Cancer 2009, 45, 228. 19097774 10.1016/j.ejca.2008.10.026

