# Supplementary material for: Plasma DNMT1 Activity for Assessing Tumor Burden and Predicting Neoadjuvant Therapy Response in Breast Cancer
Source: Adv Sci (Weinh). 2025 May 2;12(31):2501064. doi: 10.1002/advs.202501064 (PMC12376642; doi:10.1002/advs.202501064)
Supplement: Supplementary file 1 — Supporting Information [file ADVS-12-2501064-s001.docx]

Supporting Information

Plasma DNMT1 Activity for Assessing Tumor Burden and Predicting Neoadjuvant Therapy Response in Breast Cancer

Yingran Wang,^1†^ Guozhi Zhang,^2†^ Zhizhao Zhang,^2†^ Mengsi Zhang,^3^ Jiao Chen,^1^ Ke Wang,^1^ Lu Liu,^1^ Jing Bao,^1^ Ming Chen, ^1*^ Xiaowei Qi,^2*^ Mingxuan Gao^1*^

^1^ Department of Clinical Laboratory Medicine, Southwest Hospital, Third Military Medical University (Army Medical University), Chongqing, 400038, P. R. China.

^2^ Department of Breast and Thyroid Surgery, Southwest Hospital, Third Military Medical University (Army Medical University), Chongqing, 400038, P. R. China.

^3^ Institute of Pathology and Southwest Cancer Center, Southwest Hospital, Third Military Medical University (Army Medical University), Chongqing, 400038, P. R. China.
^†^ These authors contributed equally

*Correspondence: mingxuan_gao@tmmu.edu.cn; qxw9908@tmmu.edu.cn; chenming1971@tmmu.edu.cn.

**Table of Contents**

Reagents and apparatus 3

Experimental Section 4

Table S1. Price list details for completing one experiment per sample. 6

Table S2. Nucleic acid sequence design of DNMT1. 7

Table S3. Nucleic acid sequence design of M.SssI. 9

Table S4. Basic characteristics and DNMT1 protein expression of patients in different efficacy groups. 10

Figure S1. Optimization for the concentration of the substrates and primers. 11

Figure S2. TA clone sequencing results for the cytosine to thymine changes, from up to bottom: 12

Figure S3. The optimization of the reaction condition for the detection of DNMT1 by DIVA. 13

Figure S4. the optimization of the reaction condition for the detection of M.SssI by DIVA. 14

Figure S5. The In vitro Detection of M.SssI Activity using DIVA. 15

Figure S6. The heatmap of the Δcycle of DNMT1 between patients with mastitis (n = 6), healthy individuals (n = 6) and stage IV breast cancer patients (n = 6). 16

Figure S7. The IHC result of DNMT1 for the breast cancer patient. The DNMT1 staining was observed in the cytoplasm. 17

Figure S8. Identification of exosomes from four different breast cancer cell lines. 18

Figure S9. The timeline of the therapeutic regimens, ultrasound image and DNMT1 detections for patients in CR group. 19

Figure S10. The timeline of the therapeutic regimens, ultrasound image and DNMT1 detections for patients in CR group. 20

Figure S11. The timeline of the therapeutic regimens, ultrasound image and DNMT1 detections for patients in PR group. 21

Figure S12. The timeline of the therapeutic regimens, ultrasound image and DNMT1 detections for patients in PR group. 22

Figure S13. The timeline of the therapeutic regimens, ultrasound image and DNMT1 detections for patients in SD group. 23

Figure S14. The timeline of the therapeutic regimens, ultrasound image and DNMT1 detections for patients in SD group. 24

Figure S15. The timeline of the therapeutic regimens, ultrasound image and DNMT1 detections for patients in PD group. 25

Figure S16. The timeline of the therapeutic regimens, ultrasound image and DNMT1 detections for patients in PD group. 26

# Reagents and apparatus

The reagents and solutions used in this work are detailed in Supplementary materials. All DNA sequences were synthesized and purified by Sangon Biotech. Co. Ltd. (Shanghai, China). The human DNMTs (DNMT1 (# M0230), DNMT3A (# M0229), DNMT3B (# M0232)) and M.SssI methyltransferases (# M0226), *Afu* uracil-DNA glycosylase (# M0279), endonuclease IV (# M0304) and the corresponding buffer solution were obtained from New England Biolabs (Beijing, China). Methyl donor SAM was purchased from Sigma-Aldrich (St. Louis, USA). All other reagents were applied in analytical grade and were purchased from Solarbio (Beijing, China). DNA bisulfite conversion kit was supplied by Tiangen Biotech. Co. Ltd. (Beijing, China). NSC232003, hinokitiol and DC_05 inhibitors were obtained from MedChemExpress (New Jersey, USA). Normal goat serum and rabbit anti-human-DNMT1 antibody were purchased from Biotech. Co. Ltd. (Wuhan, China) and Cell Signaling Technology (Massachusetts, USA), respectively. Exosomal marker proteins (Calnexin, CD9, TSG101) were purchased from Abcam (Cambridge, Britain). The anti-mouse/rabbit polymer kit was supplied by Dako (Denmark). All solutions for the reaction were prepared with ultrapure water which was purified by a Milli-Q water purification system (18.2 MΩ cm^-1^).

RT-qPCR assay was conducted on a CFX96 Real-Time System (Bio-Rad, CA, USA). Images of PAGE were captured and analyzed on the ChemiDoc MP imaging system (Bio-Rad, CA, USA). The TEM images of breast cancer cells exosomes were conducted on a JEM-1400 Plus transmission electron microscope (JEOL, Japan).

# Experimental Section

**Development of DIV****A:** The DIVA system comprises three modules: DNA methylation, cytosine deamination, and truncation modules. For the first module, we added 1 μL of 260 fM P and N template sequences performed denaturation at 95°C for 3 min, and cooled to room temperature. We then sequentially added 2.5 μL of 1X DNMT1 buffer, 2.5 μL of 1600 μM SAM, 5 μL of 1 mg/mL BSA, and 0.4 μL of DNMT1 or 1 μL of the plasma samples, bringing the total volume to 25 μL with deionized water. The mixture was incubated at 37°C for 1 h, followed by inactivation at 65°C for 20 min, and stored at 4 °C. The second module was completed by using a commercial bisulfite conversion kit according to the kit recommendations. For the final module, we combined 10 μL of the previous modules’ products with 2 μL of 10X CS buffer, 1 μL of 200 U/mL *Afu* UDG, and 1 μL of 1000 U/mL Endo IV, bringing the total volume to 20 μL with deionized water. This mixture was incubated at 65°C for 1 h, followed by heat inactivation at 85 °C for 20 min, and stored at 4 °C.

**RT-qPCR:** Five serial dilutions of the above products with deionized water at 100-fold intervals were carried out. For a 20 μL PCR reaction, we combined 2 μL of diluted products, 0.6 μL each of F and R primer sequences, 10 μL of 2X PCR premix, and 6.8 μL of deionized water. Using a CFX96 Real-Time System, we ran PCR according to the manufacturer’s protocol: activation at 95 °C for 2 min; denaturation at 95 °C for 10 s, annealing at 37 °C for 20 s, extension at 60 °C for 25 s, for 40 cycles. The activity of DNMT1 was quantified based on the number of cycles (CT).

**Inhibition of DNMT1 activity studies:** To investigate the inhibitory effects of NSC232003, hinokitiol, and DC_05 on DNMT1 activity, we mixed 1 μL of 10^-3^ U/mL DNMT1 with different concentrations of each inhibitor, and pre-incubated the mixture in 1X DNMT1 buffer at 37 °C for 30 min. We then added 2.5 μL of 1600 μM SAM and 5 μL of 1mg/mL BSA to the reaction mixture, bringing the total volume to 25 μL with deionized water, and incubated it at 37 °C for 1 h followed by 65°C for 20 min. After bisulfite conversion, we added 2 μL of 10X CS buffer, 1 μL of 200 U/mL *Afu* UDG, and 1 μL of 1000 U/mL Endo IV, bringing the total volume to 20 μL with deionized water. The mixture underwent another incubation at 65 °C for 1 h, followed by heat inactivation at 85 °C for 20 min. Finally, the fluorescence signal was measured as described above, and the relative activity (RA) of DNMT1 was calculated using thge following equation:

$$RA= \frac{F_{i}-F_{0}}{F_{t}-F_{0}}$$

where *F*_i_, *F*_t_, and *F*_0_ represent the fluorescence intensity in the presence of tested inhibitors, in the absence of inhibitors, and in the absence of DNMT1, respectively.

**Detection of plasma DNMT1 activity levels in clinical samples:** In total, we collected 150 fresh plasma samples, comprising 35 from healthy individuals, 15 from fibroadenoma patients, and 100 from breast cancer patients at Southwest Hospital. All blood samples were centrifuged at 3500 rpm for 5 min immediately after collection and were suspended into fresh RNase-free tubes and stored at −80 °C until analysis. For each patient, we collected 5 mL plasma samples on the examination day, and another 5 mL separately at 1 month and 3 months later, totaling 15 mL. DNMT1 activity assays were performed as described above.

**Immunohistochemistry of breast cancer microarrays:** Paraffin-embedded tissues were sectioned, deparaffinized, and rehydrated through an alcohol series, followed by antigen retrieval with Tris-EDTA buffer. Then endogenous peroxidase activity was blocked using 3% H_2_O_2_ for 30 min. The slides were blocked using 10 % normal goat serum at room temperature for 1 h, and then incubated overnight with rabbit anti-human-DNMT1 antibody (diluted 1:200) at 4°C. After rinsing with wash buffer, sections were incubated with an anti-mouse/rabbit polymer kit (Envision Plus) for 30 min at room temperature. The next day, IHC samples were used for secondary antibody incubations for 30 min at room temperature, followed by DAB detection. Stained sections were observed and captured using an epifluorescence microscope.

Peritumor tissues were also collected and analyzed as controls. The IHC scores were calculated based on the percentage of positively stained cells (area score) and staining intensity. The staining intensity was scored according to the intensity of positive DNMT1 reaction. The total score was calculated using the formula: Score = intensity score × area score. The scoring of the specimens was performed using images of tumor sections by two independent pathologists.

# Table S1. Price list details for completing one experiment per sample.

| **Reagent** | **Total Price** | **Total Times** | **Cost per Reaction** |
| --- | --- | --- | --- |
| **P-strand** | 140 USD | 300 times | 0.47 USD |
| **N-strand** | 140 USD | 300 times | 0.47 USD |
| **SAM** | 32 USD | 200 times | 0.16 USD |
| **BSA** | 25 USD | 100 times | 0.25 USD |
| **DNMT1** | 169 USD | 1000 times | 0.17 USD |
| **Bisulfite Conversion Kit** | 40 USD | 100 times | 0.40 USD |
| **Afu UDG** | 107 USD | 500 times | 0.21 USD |
| **Endonuclease IV** | 108 USD | 500 times | 0.22 USD |
| **Primer** | 20 USD | 300 times | 0.07 USD |
| **2×mix** | 60 USD | 500 times | 0.12 USD |
| Total: | | | 1.53 USD |

# Table S2. Nucleic acid sequence design of DNMT1.

| **Name** | **Sequences (5’ — 3’)** | **Bases** |
| --- | --- | --- |
| **P-strand-Met-1** | /i5MedC/ATA/i5MedC/T/i5MedC/AATT/i5MedC/ATA/i5MedC/ATA/i5MedC/ATA/i5MedC/AT/i5MedC/TA/i5MedC/AT/i5MedC/TTAT/i5MedC/ATTAT/i5MedC/GTTTATCGTA/i5MedC/TATT/i5MedC/ATTATT/i5MedC/TTATTA/i5MedC/ATAT/i5MedC/TTAT/i5MedC/TAT ^a^ | 87 |
| **N-strand-Met-1** | ATAGATAAGATATGTAATAAGAATAATGAATAGTA/i5MedC/GATAAACGATAATGATAAGATGTAGATGTATGTATGTATGAATTGAGTATG | 87 |
| **P-strand-Met-2** | /i5MedC/ATA/i5MedC/T/i5MedC/AATT/i5MedC/ATA/i5MedC/ATA/i5MedC/ATA/i5MedC/AT/i5MedC/TA/i5MedC/AT/i5MedC/TTAT/i5MedC/ATTCGCGTTTA/i5MedC/G/i5MedC/GTATTA/i5MedC/TATT/i5MedC/ATTATT/i5MedC/TTATTA/i5MedC/ATAT/i5MedC/TTAT/i5MedC/TAT | 91 |
| **N-strand-Met-2** | ATAGATAAGATATGTAATAAGAATAATGAATAGTAATACGCGTAAA/i5MedC/G/i5MedC/GAATGATAAGATGTAGATGTATGTATGTATGAATTGAGTATG | 91 |
| **P-strand-Met-3** | /i5MedC/ATA/i5MedC/T/i5MedC/AATT/i5MedC/ATA/i5MedC/ATA/i5MedC/ATA/i5MedC/AT/i5MedC/TA/i5MedC/AT/i5MedC/TTAT/i5MedC/ATTCGCGCGTATA/i5MedC/G/i5MedC/G/i5MedC/GTTA/i5MedC/TATT/i5MedC/ATTATT/i5MedC/TTATTA/i5MedC/ATAT/i5MedC/TTAT/i5MedC/TAT | 93 |
| **N-strand-Met-3** | ATAGATAAGATATGTAATAAGAATAATGAATAGTAACGCGCGTATA/i5MedC/G/i5MedC/G/i5MedC/GAATGATAAGATGTAGATGTATGTATGTATGAATTGAGTATG | 93 |
| **P-strand-Met-4** | /i5MedC/ATA/i5MedC/T/i5MedC/AATT/i5MedC/ATA/i5MedC/ATA/i5MedC/ATA/i5MedC/AT/i5MedC/TA/i5MedC/AT/i5MedC/TTAT/i5MedC/ATTATCCGGTATA/i5MedC//i5MedC/GGTTA/i5MedC/TATT/i5MedC/ATTATT/i5MedC/TTATTA/i5MedC/ATAT/i5MedC/TTAT/i5MedC/TAT | 91 |
| **N-strand-Met-4** | ATAGATAAGATATGTAATAAGAATAATGAATAGTAACCGGTATA/i5MedC//i5MedC/GGATAATGATAAGATGTAGATGTATGTATGTATGAATTGAGTATG | 91 |
| **P-strand-Met-5** | /i5MedC/ATA/i5MedC/T/i5MedC/AATT/i5MedC/ATA/i5MedC/ATA/i5MedC/ATA/i5MedC/AT/i5MedC/TA/i5MedC/AT/i5MedC/TTAT/i5MedC/ATTAT/i5MedC/GTCGTTATTA/i5MedC/TATT/i5MedC/ATTATT/i5MedC/TTATTA/i5MedC/ATAT/i5MedC/TTAT/i5MedC/TAT | 87 |
| **N-strand-Met-5** | ATAGATAAGATATGTAATAAGAATAATGAATAGTAATAA/i5MedC/GACGATAATGATAAGATGTAGATGTATGTATGTATGAATTGAGTATG | 87 |
| **P-strand-Met-6** | /i5MedC/ATA/i5MedC/T/i5MedC/AATT/i5MedC/ATA/i5MedC/ATA/i5MedC/ATA/i5MedC/AT/i5MedC/TA/i5MedC/AT/i5MedC/TTAT/i5MedC/ATTAT/i5MedC/GTTTCGATTA/i5MedC/TATT/i5MedC/ATTATT/i5MedC/TTATTA/i5MedC/ATAT/i5MedC/TTAT/i5MedC/TAT | 87 |
| **N-strand-Met-6** | ATAGATAAGATATGTAATAAGAATAATGAATAGTAAT/i5MedC/GAAACGATAATGATAAGATGTAGATGTATGTATGTATGAATTGAGTATG | 87 |
| **P-strand-Met-7** | /i5MedC/ATA/i5MedC/T/i5MedC/AATT/i5MedC/ATA/i5MedC/ATA/i5MedC/ATA/i5MedC/AT/i5MedC/TA/i5MedC/AT/i5MedC/TTAT/i5MedC/ATTAT/i5MedC/GTTTATTACG/i5MedC/TATT/i5MedC/ATTATT/i5MedC/TTATTA/i5MedC/ATAT/i5MedC/TTAT/i5MedC/TAT | 87 |
| **N-strand-Met-7** | ATAGATAAGATATGTAATAAGAATAATGAATAG/i5MedC/GTAATAAACGATAATGATAAGATGTAGATGTATGTATGTATGAATTGAGTATG | 87 |
| **F-Primer** | ATAGATAAGATATGTAATAAG | 21 |
| **R-Primer** | CATACTCAATTCATACATACA | 21 |

^a^ /i5MedC/ represents 5-methyl cytosine.

# Table S3. Nucleic acid sequence design of M.SssI.

| **Name** | **Sequences (5’ — 3’)** | **Bases** |
| --- | --- | --- |
| **P-strand-Met-1** | i5MedC/ATA/i5MedC/T/i5MedC/AATT/i5MedC/ATA/i5MedC/ATA/i5MedC/ATA/i5MedC/AT/i5MedC/TA/i5MedC/AT/i5MedC/TTAT/i5MedC/ATTCGCGTTTATATTA/i5MedC/TATT/i5MedC/ATTATT/i5MedC/TTATTA/i5MedC/ATAT/i5MedC/TTAT/i5MedC/TAT | 87 |
| **N-strand-1** | ATAGATAAGATATGTAATAAGAATAATGAATAGTAATATAAACGCGAATGATAAGATGTAGATGTATGTATGTATGAATTGAGTATG | 87 |
| **P-strand-Met-2** | i5MedC/ATA/i5MedC/T/i5MedC/AATT/i5MedC/ATA/i5MedC/ATA/i5MedC/ATA/i5MedC/AT/i5MedC/TA/i5MedC/AT/i5MedC/TTAT/i5MedC/ATTATCGTTTATCGTA/i5MedC/TATT/i5MedC/ATTATT/i5MedC/TTATTA/i5MedC/ATAT/i5MedC/TTAT/i5MedC/TAT | 87 |
| **N-strand-2** | ATAGATAAGATATGTAATAAGAATAATGAATAGTACGATAAACGATAATGATAAGATGTAGATGTATGTATGTATGAATTGAGTATG | 87 |
| **P-strand-Met-3** | i5MedC/ATA/i5MedC/T/i5MedC/AATT/i5MedC/ATA/i5MedC/ATA/i5MedC/ATA/i5MedC/AT/i5MedC/TA/i5MedC/AT/i5MedC/TTAT/i5MedC/ATTCGCGCGTATATTA/i5MedC/TATT/i5MedC/ATTATT/i5MedC/TTATTA/i5MedC/ATAT/i5MedC/TTAT/i5MedC/TAT | 87 |
| **N-strand-3** | ATAGATAAGATATGTAATAAGAATAATGAATAGTAATATACGCGCGAATGATAAGATGTAGATGTATGTATGTATGAATTGAGTATG | 87 |
| **P-strand-Met-4** | i5MedC/ATA/i5MedC/T/i5MedC/AATT/i5MedC/ATA/i5MedC/ATA/i5MedC/ATA/i5MedC/AT/i5MedC/TA/i5MedC/AT/i5MedC/TTAT/i5MedC/ATTATCCGGTATATTA/i5MedC/TATT/i5MedC/ATTATT/i5MedC/TTATTA/i5MedC/ATAT/i5MedC/TTAT/i5MedC/TAT | 87 |
| **N-strand-4** | ATAGATAAGATATGTAATAAGAATAATGAATAGTAATATACCGGATAATGATAAGATGTAGATGTATGTATGTATGAATTGAGTATG | 87 |
| **F-Primer** | ATAGATAAGATATGTAATAAG | 21 |
| **R-Primer** | CATACTCAATTCATACATACA | 21 |

# Table S4. Basic characteristics and DNMT1 protein expression of patients in different efficacy groups.

| Characteristics | CR (n=5) | PR (n=6) | SD (n=6) | PD (n=5) |
| --- | --- | --- | --- | --- |
| DNMT1^a^ (median [IQR]) | 1.82(1.36, 2.37) | 0.96 (0.81, 1.37) | 0.44 (0.16, 0.71) | -0.37 (-0.73, -0.32) |
| Age [mean (SD)], years | 42.60 [7.02] | 54.67 [6.98] | 50.83 [5.12] | 48.80 [2.95] |
| Location of the tumor, n (%) |  |  |  |  |
| Left breast | 4 (80.00) | 3 (50.00) | 2 (33.33) | 1 (20.00) |
| Right breast | 1 (20.00) | 3 (50.00) | 4 (66.67) | 4 (80.00) |
| Menopausal status, n (%) |  |  |  |  |
| Premenopausal | 4 (80.00) | 1 (16.67) | 1 (16.67) | 2 (40.00) |
| Postmenopausal | 1 (20.00) | 5 (83.33) | 5 (83.33) | 3 (60.00) |
| Pathological T stage, n (%) |  |  |  |  |
| 1 | 0 | 1 (16.67) | 0 | 2 (40.00) |
| 2 | 4 (80.00) | 4 (66.67) | 5 (83.33) | 2 (40.00) |
| 3 | 1 (20.00) | 1 (16.67) | 1 (16.67) | 1 (20.00) |
| Pathological N stage, n (%) |  |  |  |  |
| 0 | 3 (60.00) | 5 (83.33) | 4 (66.67) | 3 (60.00) |
| 1 | 2 (40.00) | 1 (16.67) | 2 (33.33) | 2 (40.00) |
| HR status, n (%) |  |  |  |  |
| Negative | 3 (60.00) | 2 (33.33) | 4 (66.67) | 5 (100.00) |
| Positive | 2 (40.00) | 4 (66.67) | 2 (33.33) | 0 |
| HER2 status, n (%) |  |  |  |  |
| 0, 1+, 2+ and FISH- | 1 (20.00) | 0 (0.00) | 2 (33.33) | 1 (20.00) |
| 3+, 2+ and FISH+ | 4 (80.00) | 6 (100.00) | 4 (66.67) | 4 (80.00) |
| Ki67 [mean (SD)] | 41.00 [19.50] | 32.50 [13.32] | 22.33 [14.88] | 33.40 [22.53] |

Abbreviations: CR, complete response; PR, partial response; SD, stable disease; PD, progressive disease; HR, hormone status; HER2, human epidermal growth factor receptor 2; FISH, fluorescence in situ hybridization; IQR, interquartile range.

^a^ Difference of DNMT1 protein in blood samples of patients before and after neoadjuvant therapy.


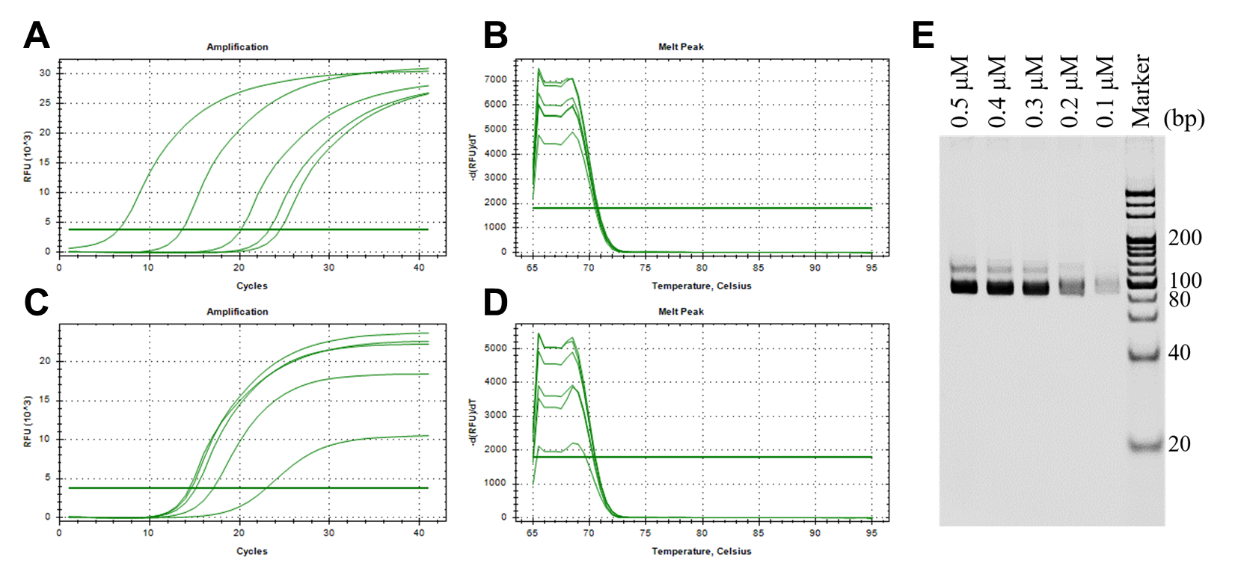


Figure S1. Optimization for the concentration of the substrates and primers. **(A)** The amplification curves for different concentrations of substrates, 200 nM, 2 nM, 20 pM, 200 fM, 2 fM, from left to right. **(B)** The corresponding melting curves for different concentrations of substrates. **(C)** The amplification curves for different concentrations of primers, 0.5 μM, 0.4 μM, 0.3 μM, 0.2 μM, 0.1 μM, from top to bottom. **(D)** The corresponding melting curves for different concentrations of primers. E) the denatured PAGE gel for for different concentrations of substrates.


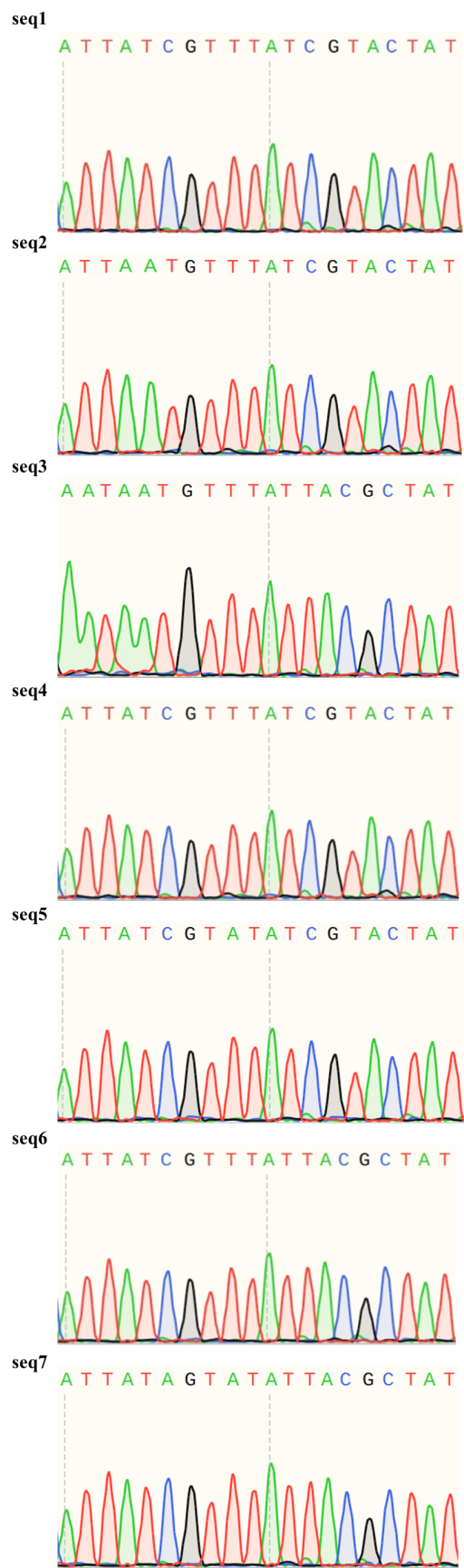


Figure S2. TA clone sequencing results for the cytosine to thymine changes, from up to bottom: DIVA, DIVA without SAM, DIVA without DNMT1, DIVA without DNMT1 and *Afu* UDG and endo IV, DIVA without DNMT1 and bisulfite, DIVA without DNMT1 and *Afu* UDG, DIVA without DNMT1 and endo IV.


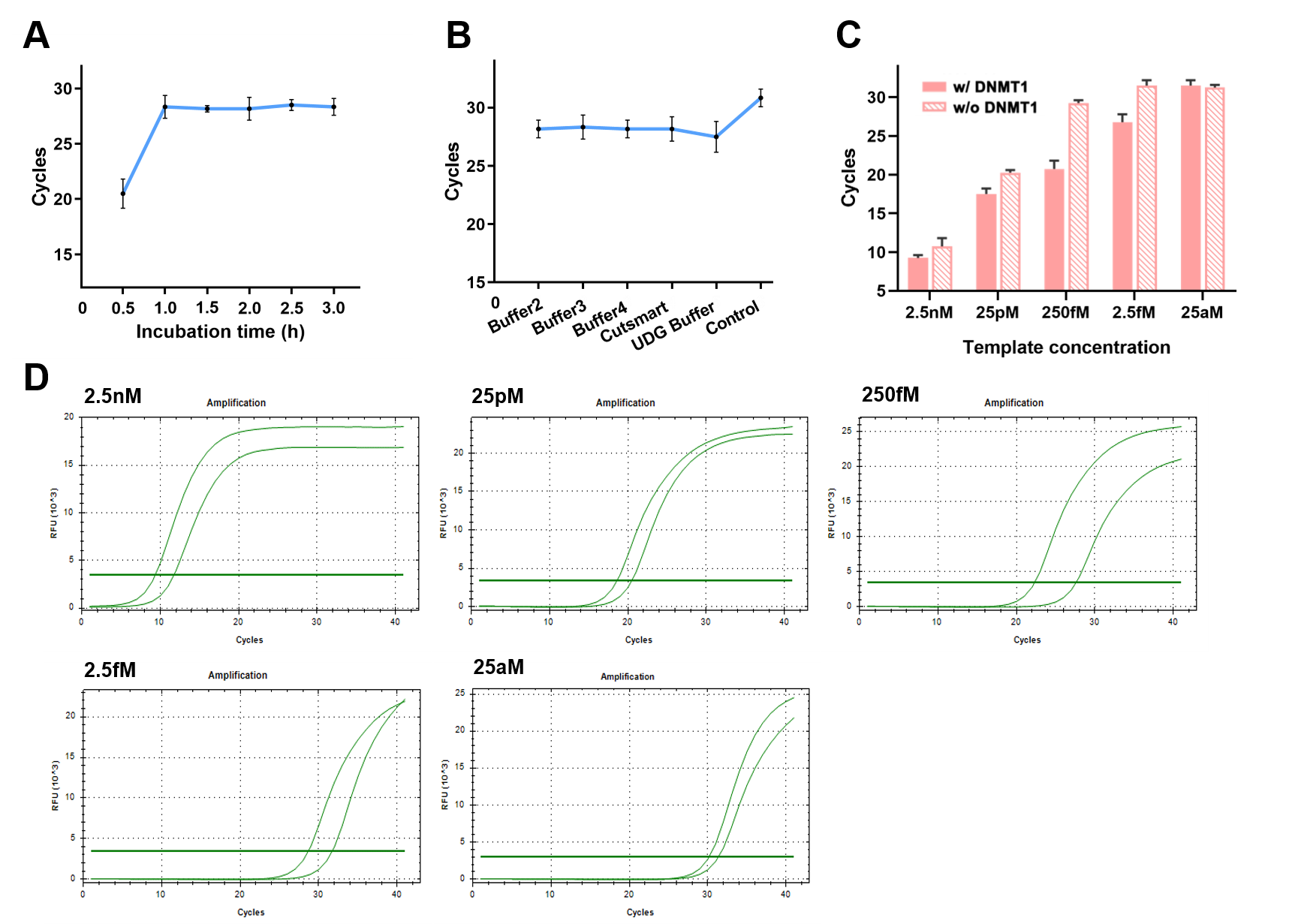


Figure S3. The optimization of the reaction condition for the detection of DNMT1 by DIVA. (**A)** the amplification cycles by incubating different time. **(B)** The amplification cycles by using different buffers, buffer 2: 50 mM NaCl, 10 mM Tris-HCl, 10 mM MgCl_2_, 1 mM DTT, pH 7.9@25°C; buffer 3: 100 mM NaCl, 50 mM Tris-HCl, 10 mM MgCl_2_, 1 mM DTT, pH 7.9@25°C;, buffer 4: 50 mM Potassium Acetate, 20 mM Tris-acetate, 10 mM Magnesium Acetate, 1 mM DTT, pH 7.9@25°C; Cutsmart: 50 mM Potassium Acetate, 20 mM Tris-acetate, 10 mM Magnesium Acetate, 100 µg/ml BSA, pH 7.9@25°C; UDG buffer: 20 mM Tris-HCl, 1 mM DTT, 1 mM EDTA, pH 8 @ 25°C; control: 137mM NaCl, 2.7mM KCl, 10mM Na_2_HPO_4_, 2mM KH_2_PO_4,_ pH 7.4. **(C)** The amplification cycles for different concentrations of substrates with or without DNMT1. **(D)** The amplification curves for different concentrations of substrates with or without DNMT1.


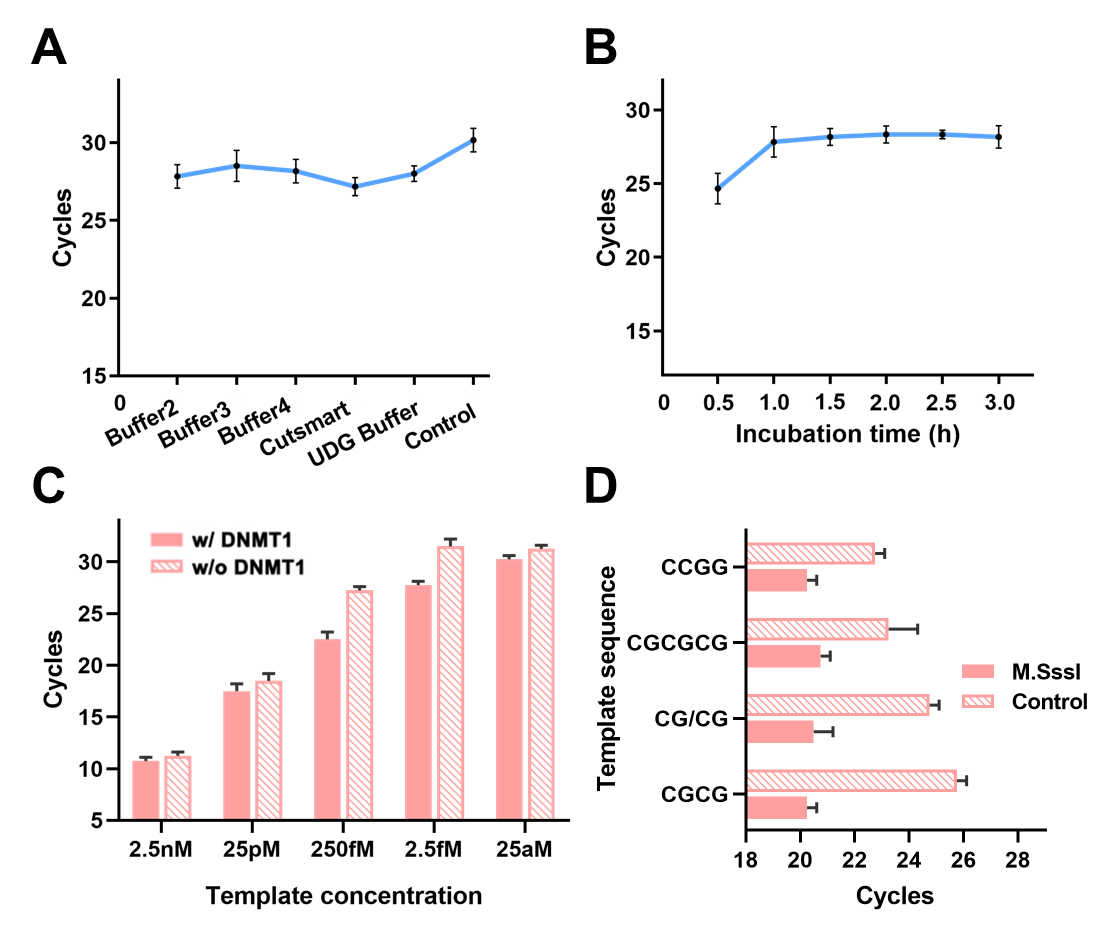


Figure S4. the optimization of the reaction condition for the detection of M.SssI by DIVA. **(A)** the amplification cycles by using different buffers, buffer 2: 50 mM NaCl, 10 mM Tris-HCl, 10 mM MgCl_2_, 1 mM DTT, pH 7.9@25°C; buffer 3: 100 mM NaCl, 50 mM Tris-HCl, 10 mM MgCl_2_, 1 mM DTT, pH 7.9@25°C;, buffer 4: 50 mM Potassium Acetate, 20 mM Tris-acetate, 10 mM Magnesium Acetate, 1 mM DTT, pH 7.9@25°C; Cutsmart: 50 mM Potassium Acetate, 20 mM Tris-acetate, 10 mM Magnesium Acetate, 100 µg/ml BSA, pH 7.9@25°C; UDG buffer: 20 mM Tris-HCl, 1 mM DTT, 1 mM EDTA, pH 8 @ 25°C; control: 137mM NaCl, 2.7mM KCl, 10mM Na_2_HPO_4_, 2mM KH_2_PO_4,_ pH 7.4. **(B)** The amplification cycles by incubating different time. **(C)** The amplification cycles for different concentrations of substrates with or without DNMT1. **(D)** The amplification cycles for different CpG patterns of substrates with or without DNMT1.


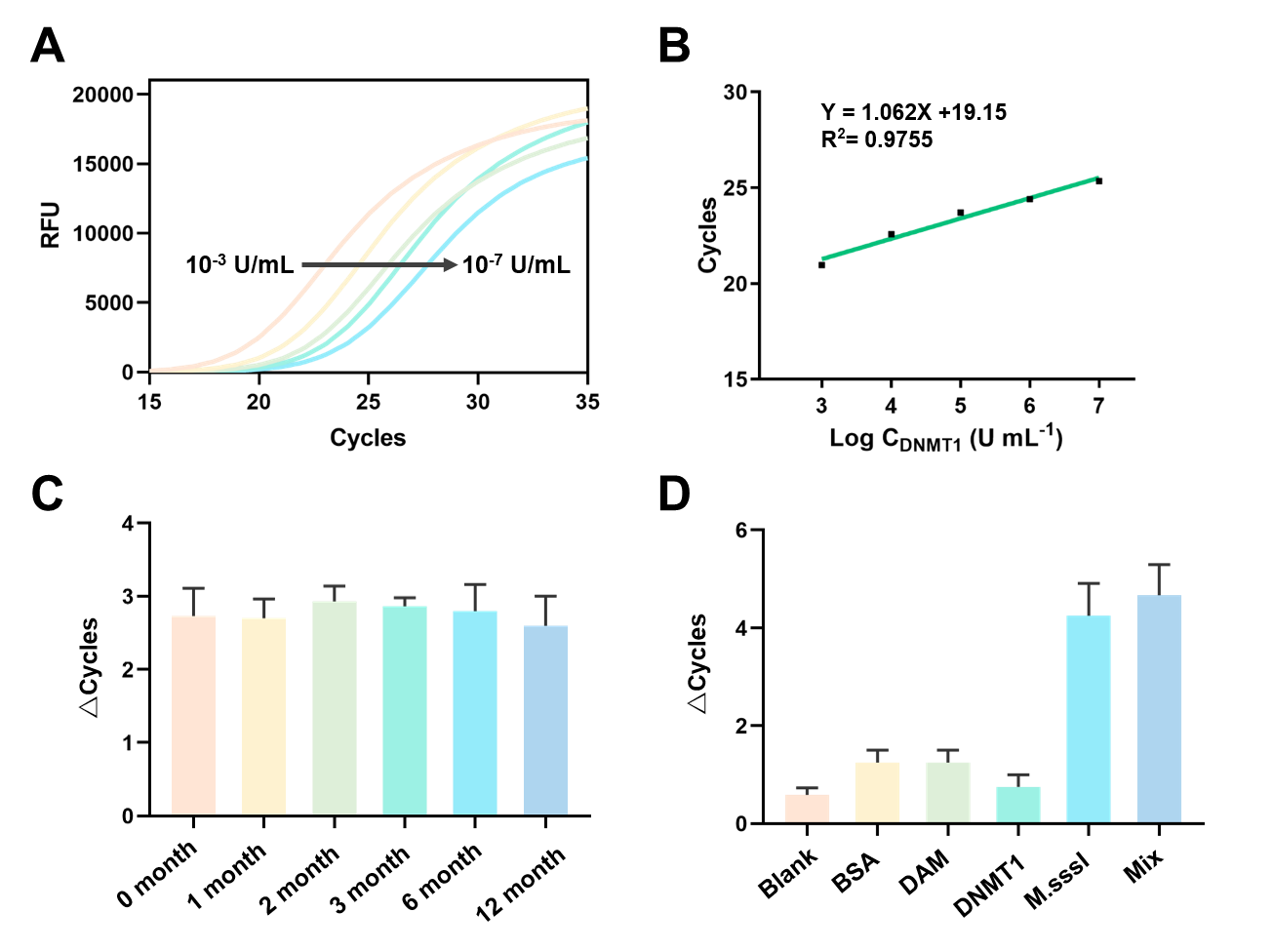


Figure S5. The In vitro Detection of M.SssI Activity using DIVA. **(A)** The *in vitro* detection range of M.SssI. **(B)** Linear regression curve for detection of M.SssI, *Δ cycle* represented the cycle difference to the negative control in the PCR process (n=3). **(C)** The stability assay tested at 0, 1, 2, 3, 6, and 12 months of M.SssI by DIVA. **(D)** The specificity assay of different substances by DIVA, *C*_BSA_ = 1mg/mL*, C*_DAM_ = 1×10^-3^ U/mL, *C*_DNMT1_ = 1×10^-3^ U/mL, *C*_M.SssI_ = 1×10^-3^ U/mL.


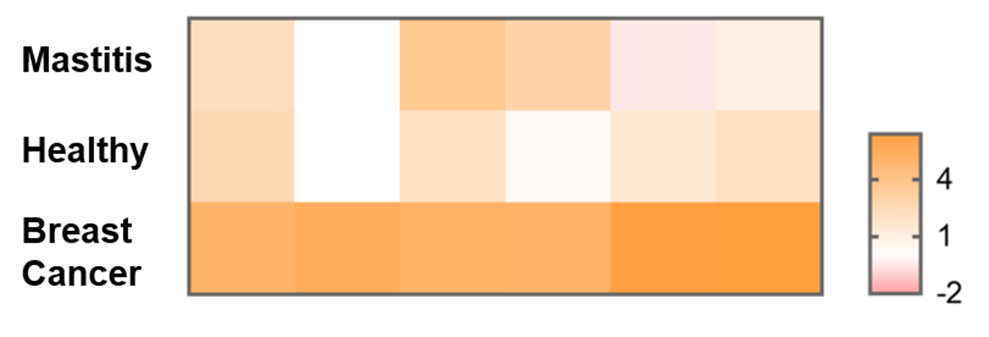


# Figure S6. The heatmap of the Δcycle of DNMT1 between patients with mastitis (n = 6), healthy individuals (n = 6) and stage IV breast cancer patients (n = 6).


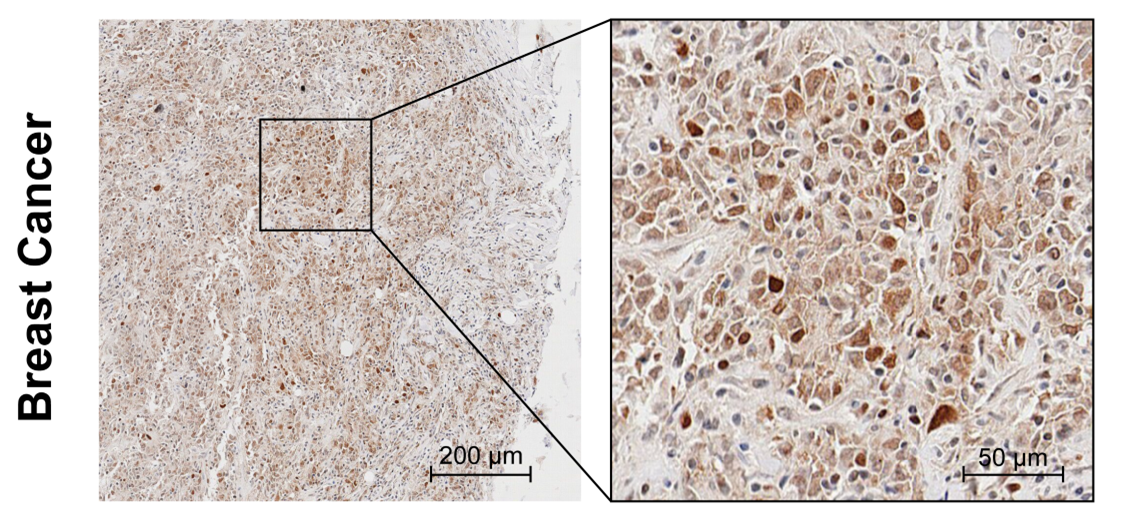


# Figure S7. The IHC result of DNMT1 for the breast cancer patient. The DNMT1 staining was observed in the cytoplasm.


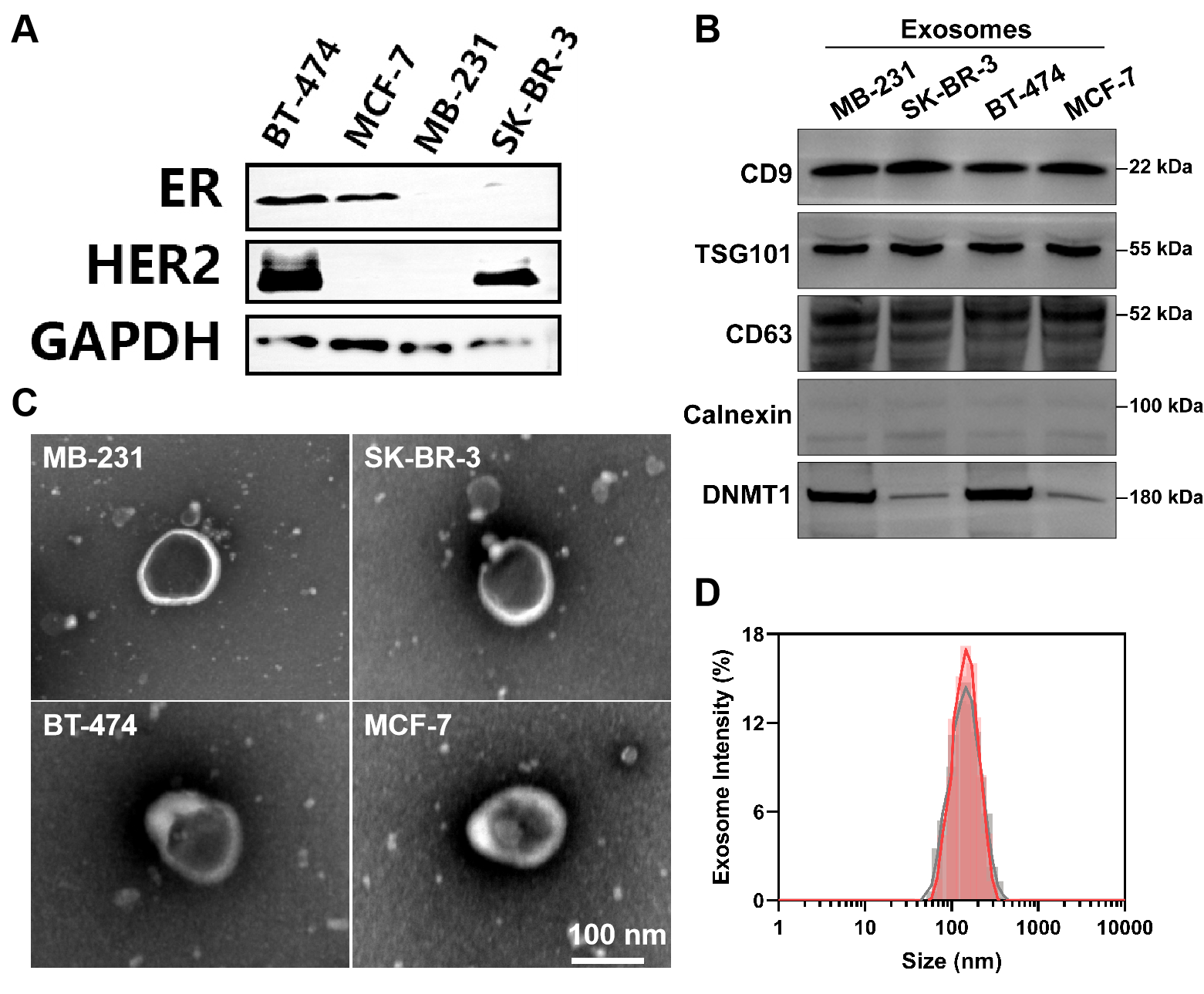


Figure S8. Identification of exosomes from four different breast cancer cell lines. (**A)** the estrogen receptor (ER) and human epidermal growth factor receptor 2 (HER2) expression on the BT-474 (ER^+^/HER2^+^), MCF-7 (ER^+^/HER2^-^), MB-231 (ER^-^/HER2^-^) and SK-BR-3 (ER^-^/HER2^+^). **(B)** the DNMT1 expression in the exosomes of four different breast cancer cell lines. (**C)** the TEM images of exosomes of four different breast cancer cell lines. (**D)** nanoparticle tracking analysis of breast cancer cell MB-231 exosomes.


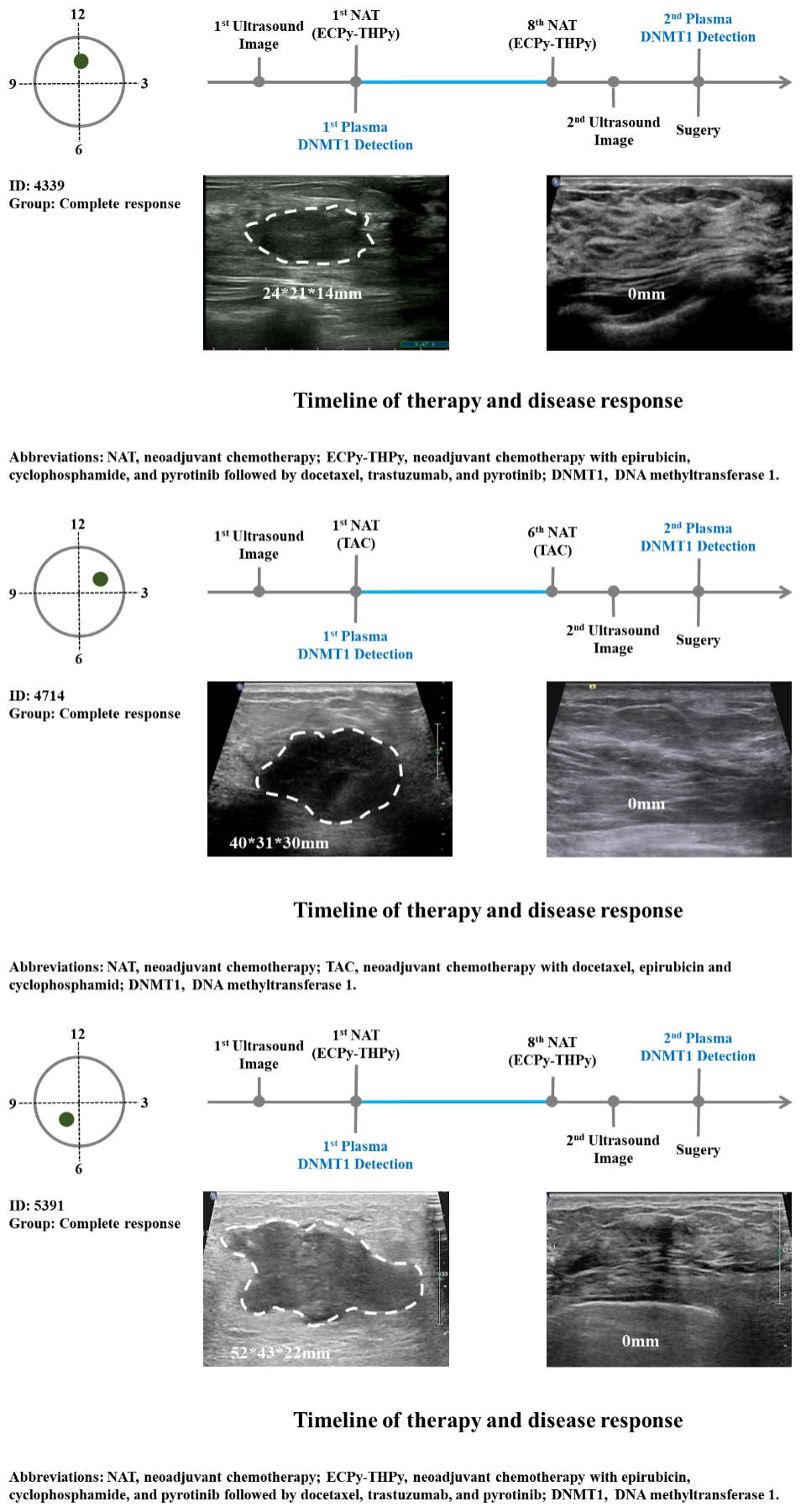


# Figure S9. The timeline of the therapeutic regimens, ultrasound image and DNMT1 detections for patients in CR group.


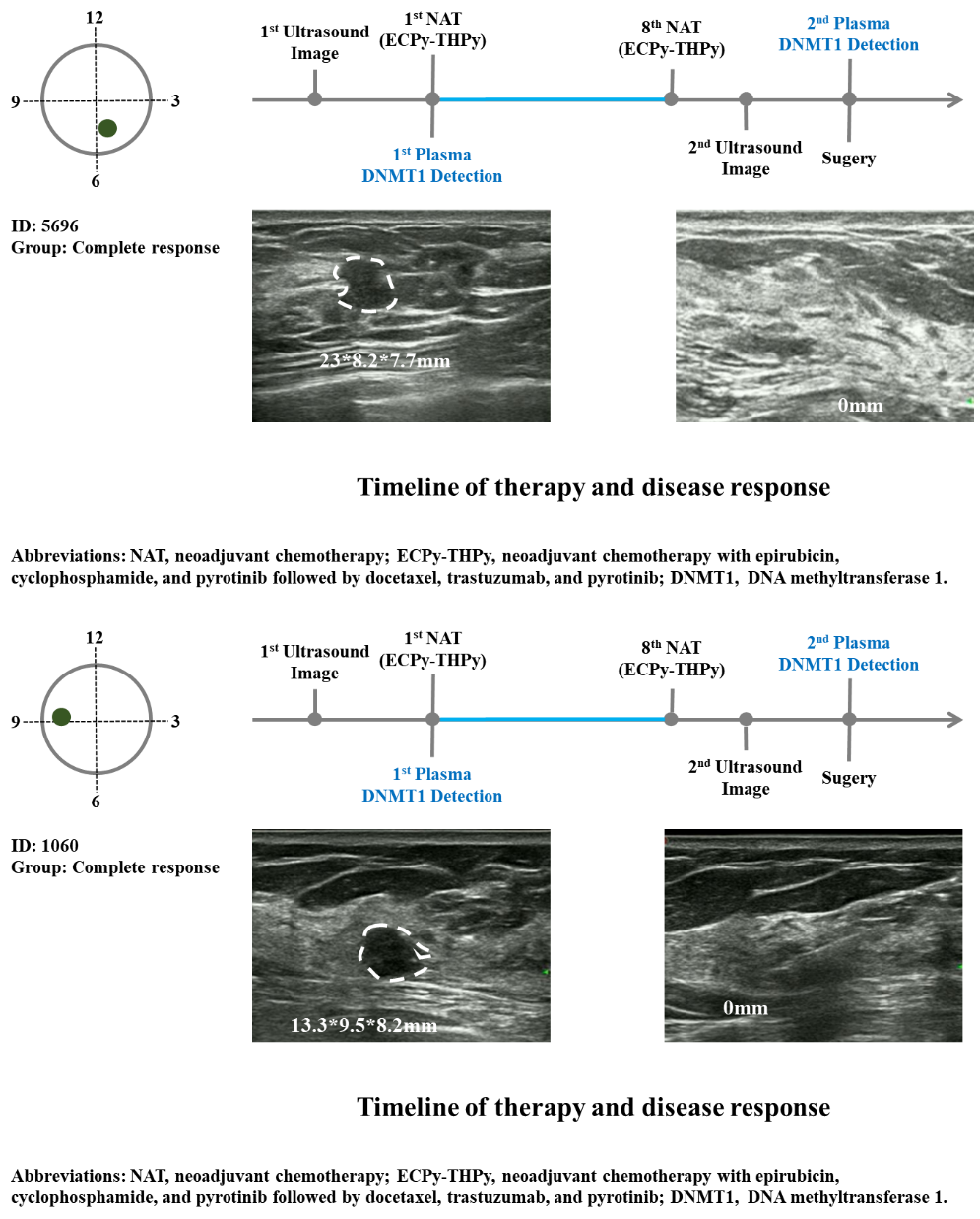


# Figure S10. The timeline of the therapeutic regimens, ultrasound image and DNMT1 detections for patients in CR group.


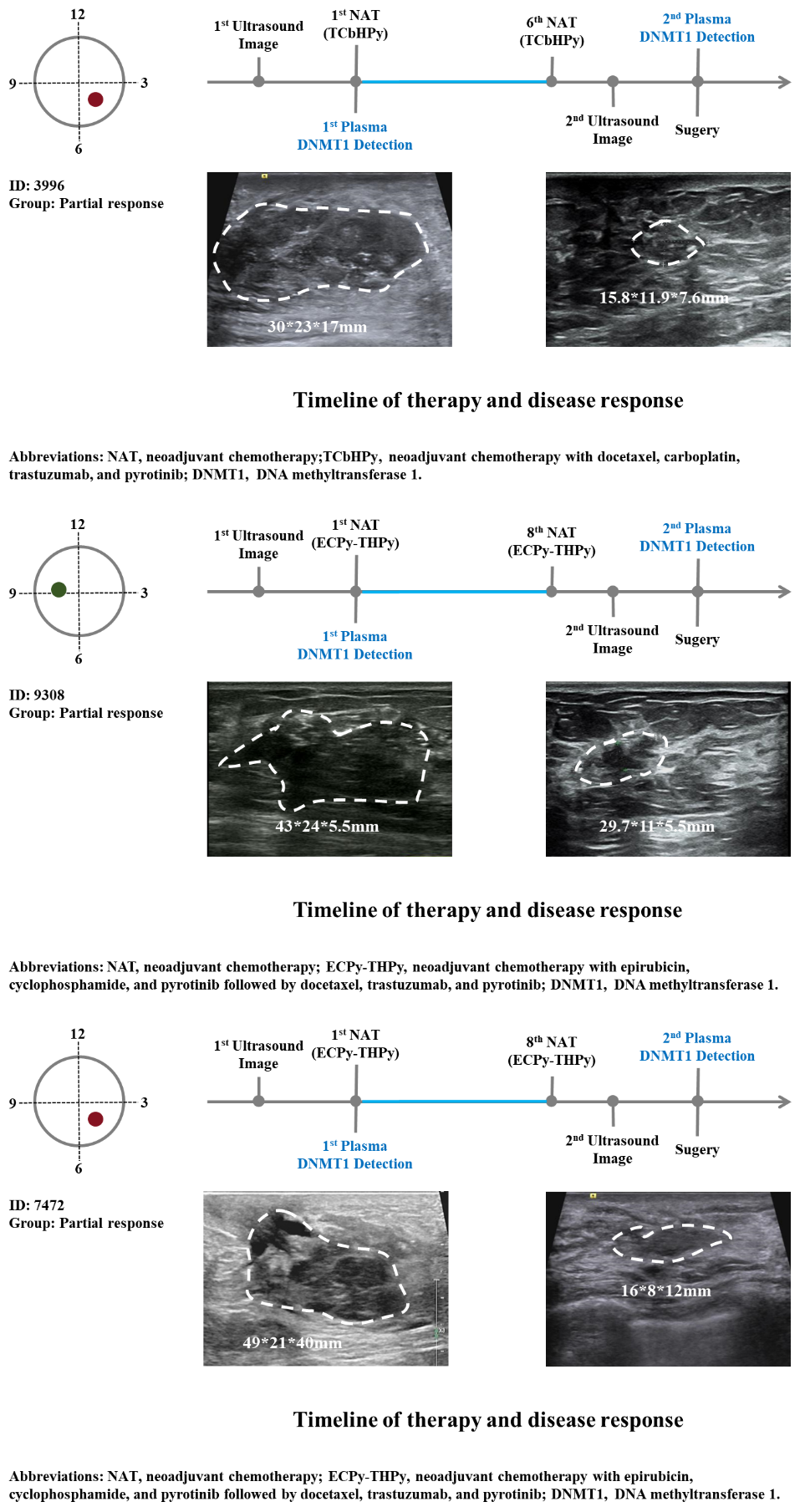


# Figure S11. The timeline of the therapeutic regimens, ultrasound image and DNMT1 detections for patients in PR group.


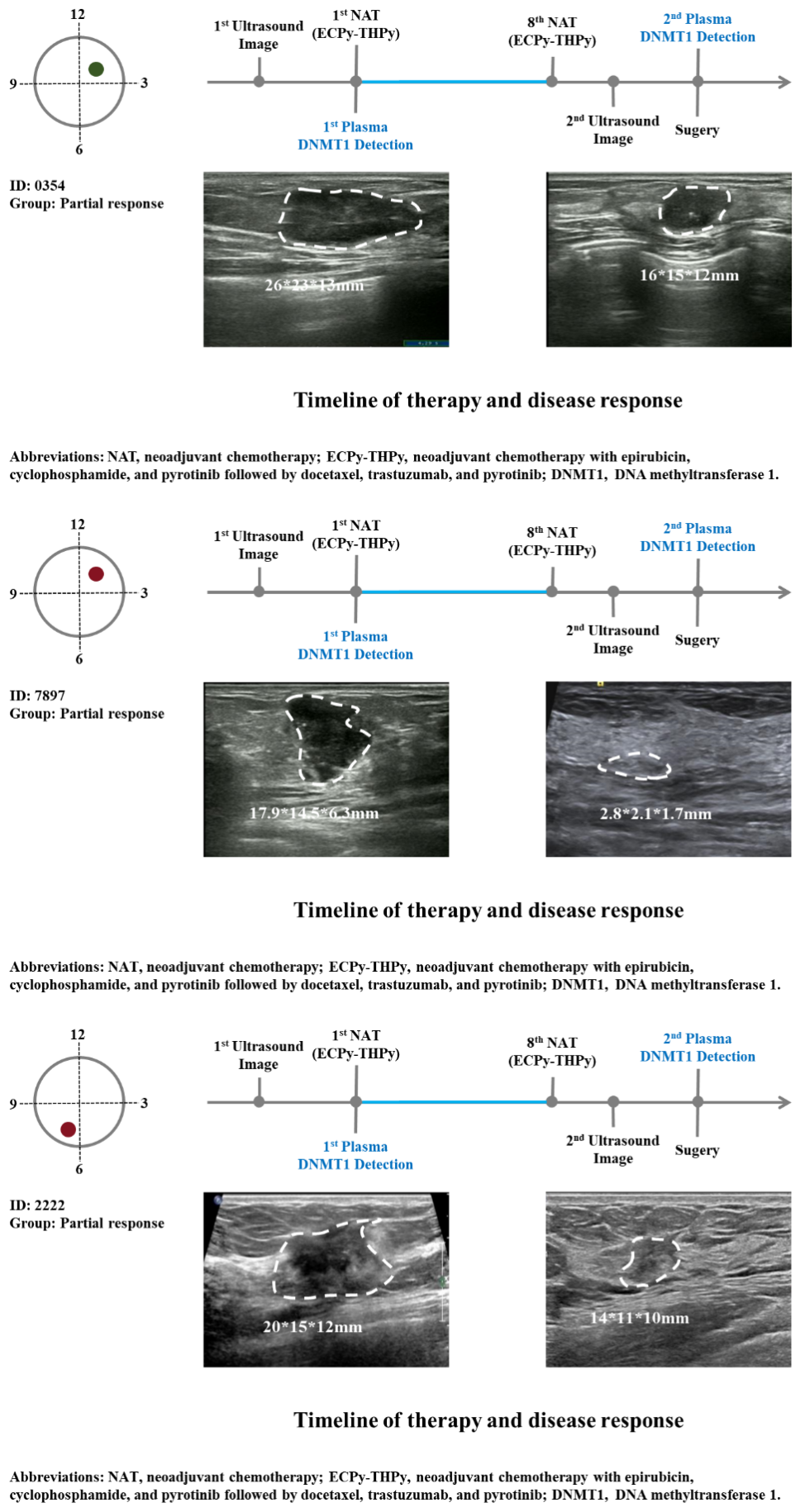


# Figure S12. The timeline of the therapeutic regimens, ultrasound image and DNMT1 detections for patients in PR group.


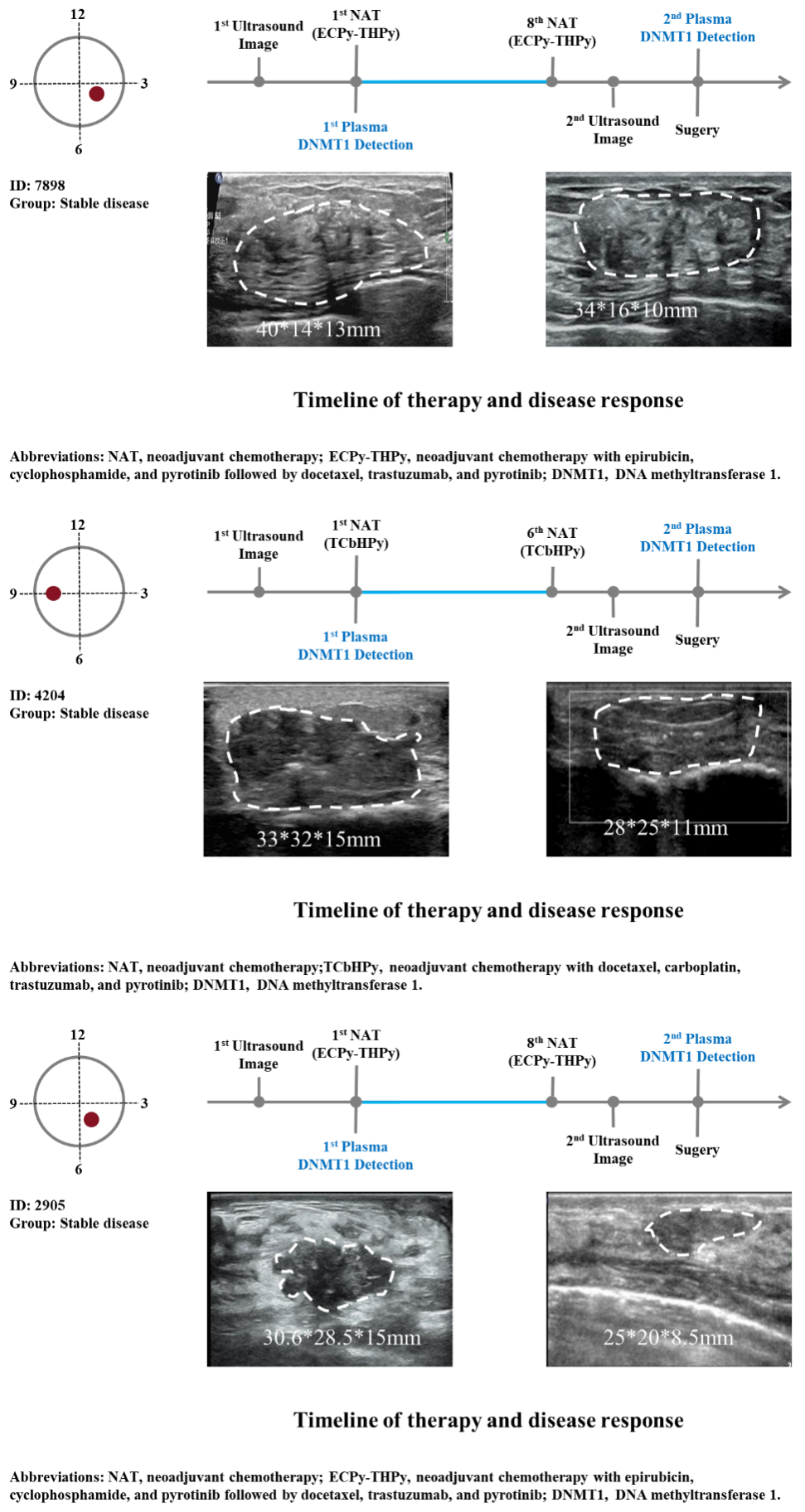


# Figure S13. The timeline of the therapeutic regimens, ultrasound image and DNMT1 detections for patients in SD group.


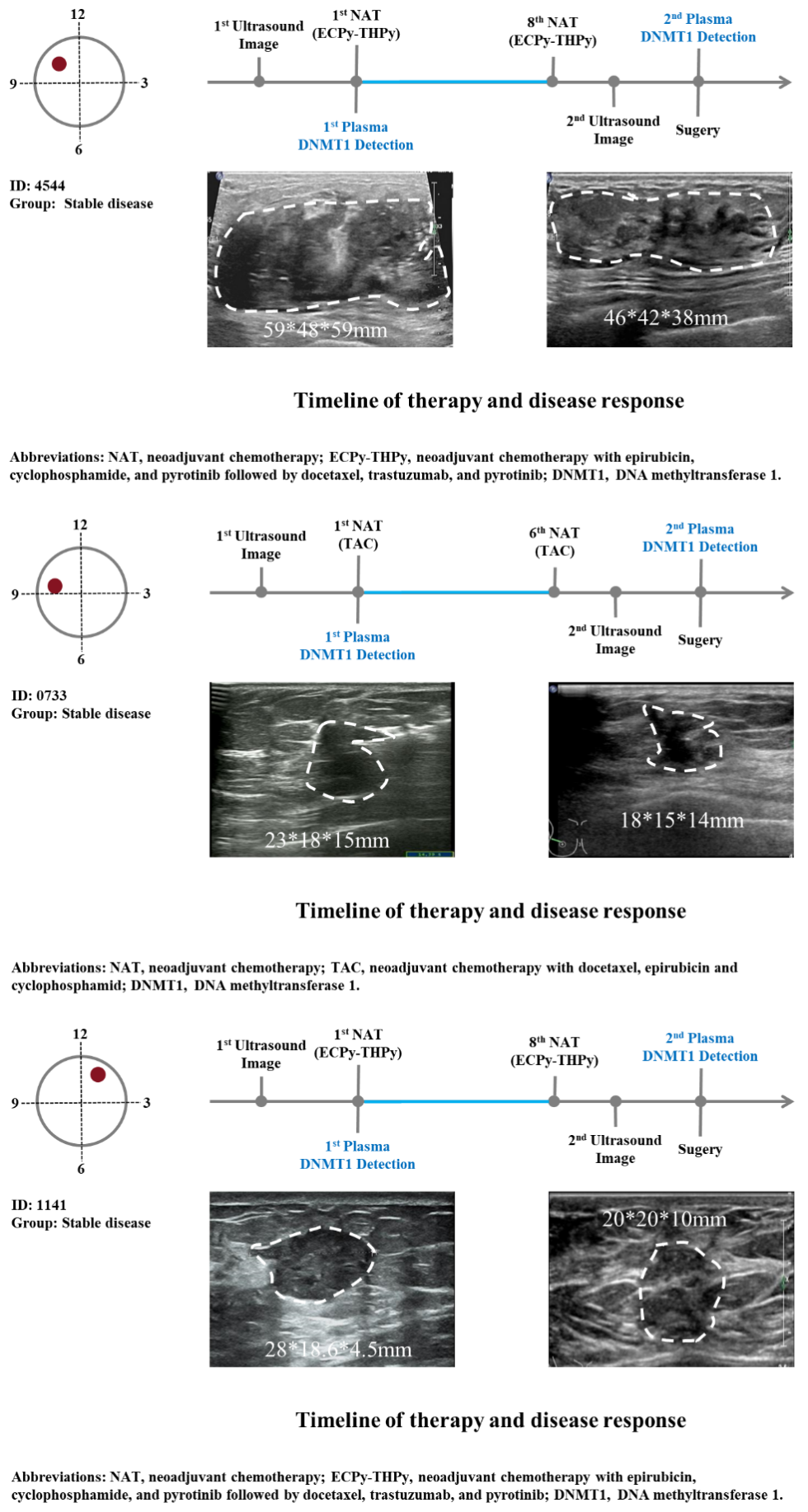


# Figure S14. The timeline of the therapeutic regimens, ultrasound image and DNMT1 detections for patients in SD group.


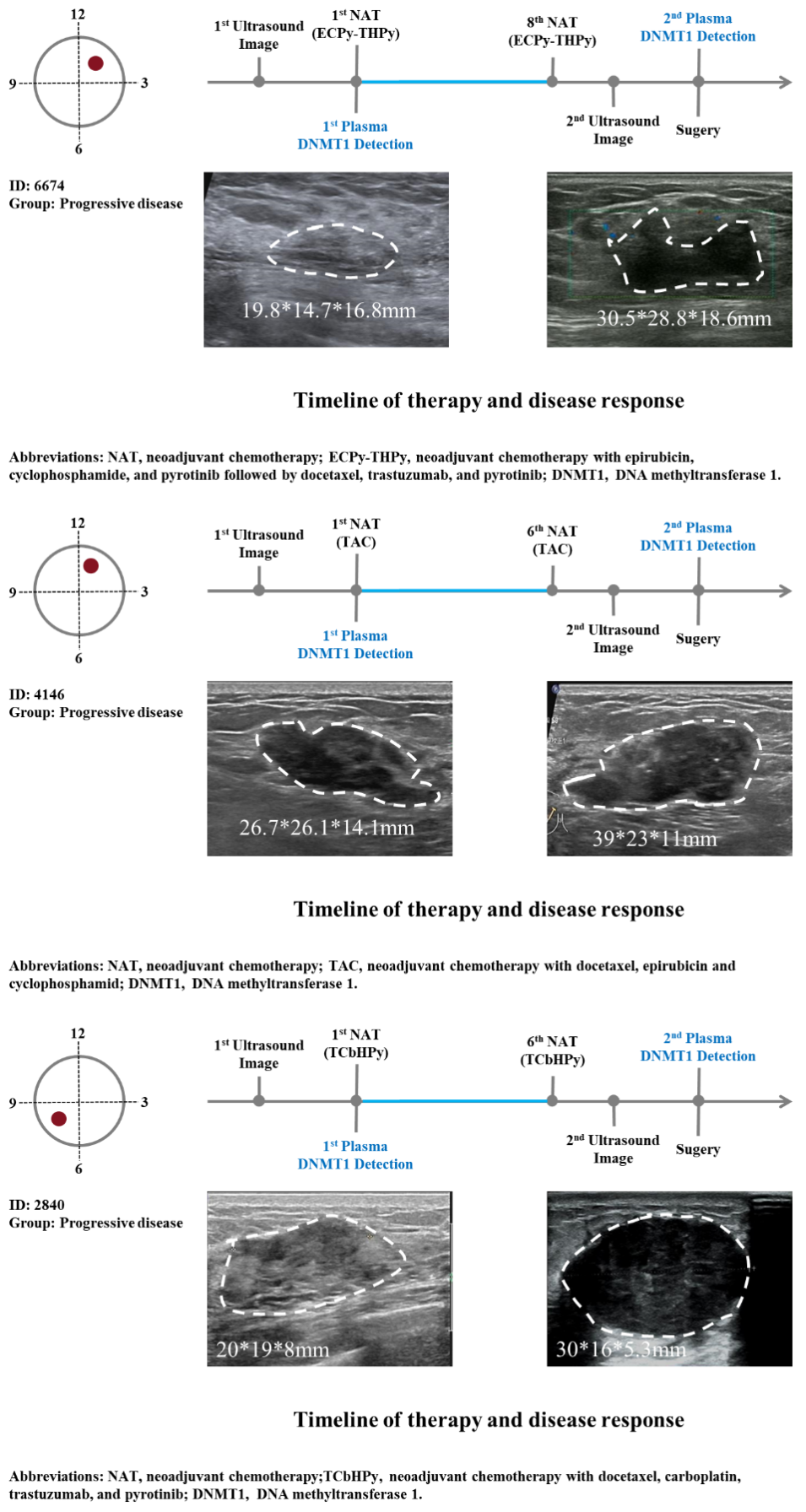


# Figure S15. The timeline of the therapeutic regimens, ultrasound image and DNMT1 detections for patients in PD group.


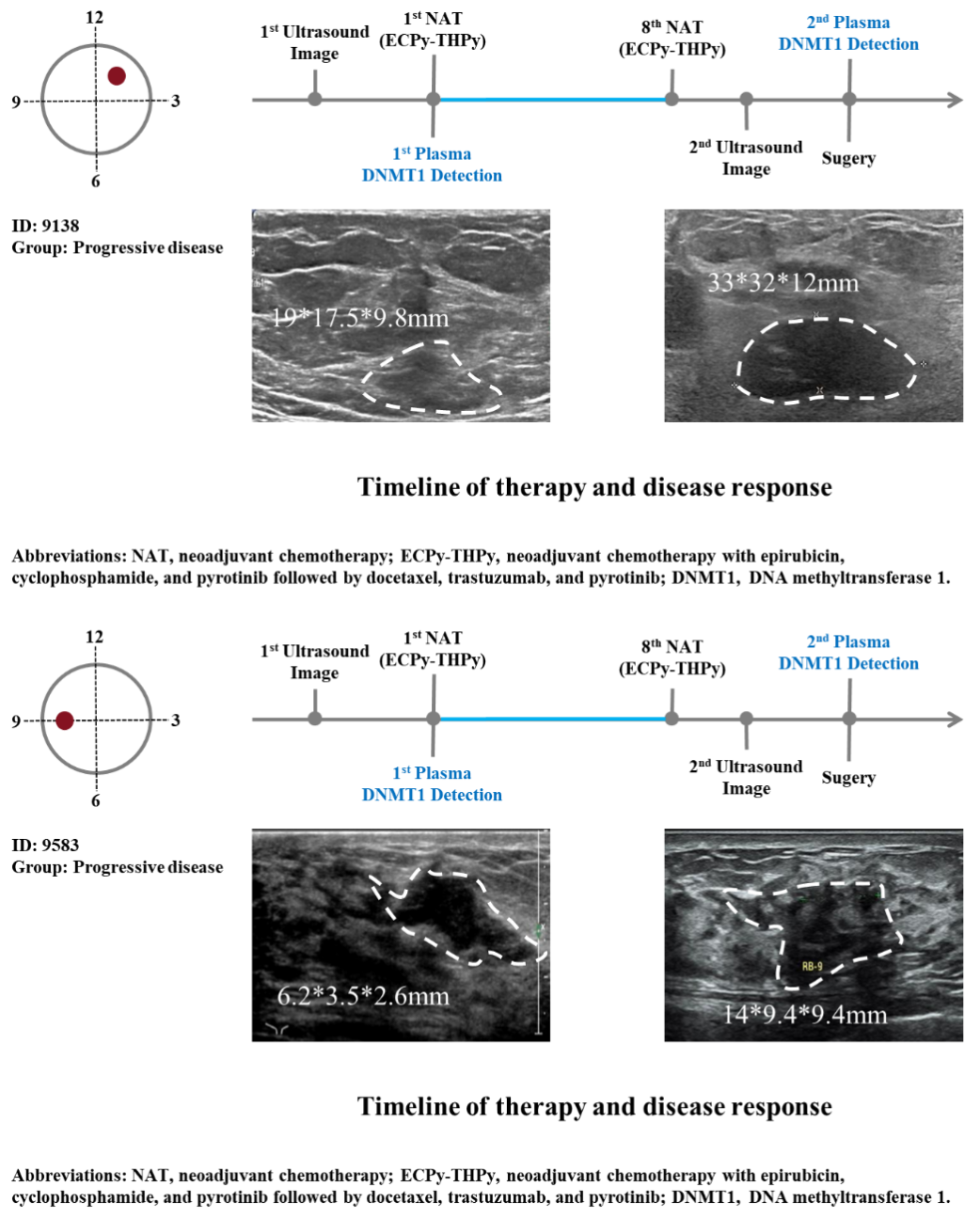


# Figure S16. The timeline of the therapeutic regimens, ultrasound image and DNMT1 detections for patients in PD group.
